# Thioester-Containing Ionizable Lipids with Enhanced Endosomal Escape and Biodegradability for mRNA and tRNA Delivery

**DOI:** 10.3390/pharmaceutics18040472

**Published:** 2026-04-13

**Authors:** Álvaro Peña, Esther Broset, Enrique Lucia, Laura García-Latorre, Víctor Navarro, Carlos Matute, Ana Gallego-Lleyda, Teresa Alejo, Eduardo Romanos, Alba García, Juan Martínez-Oliván, Javier Giménez-Warren

**Affiliations:** 1 Certest Pharma, Certest Biotec S. L., San Mateo de Gállego, 50840 Zaragoza, Spainebroset@certest.es (E.B.); elucia@certest.es (E.L.); lgarcia@certest.es (L.G.-L.); vnavarro@certest.es (V.N.); cmatute@certest.es (C.M.); agallego@certest.es (A.G.-L.); talejo@certest.es (T.A.); 2 Medical Imaging and Phenotyping Core Facility, Aragon Health Sciences Institute (IACS), 50009 Zaragoza, Spain; eromanos.iacs@aragon.es (E.R.); agarciagil.iacs@aragon.es (A.G.)

**Keywords:** RNA, thioester ionizable lipids, lipid nanoparticles (LNPs), biodegradable, tRNA

## Abstract

**Background/Objectives**: Lipid nanoparticles (LNPs) containing ionizable lipids represent the most advanced non-viral delivery vehicles and have become state-of-the-art carriers for RNA therapeutics. However, further improvements in endosomal escape efficiency and biodegradability are still needed, especially for nucleic acids with transient activity such as messenger RNA (mRNA) and transfer RNA (tRNA). **Methods**: In this study, a novel library of highly biodegradable ionizable lipids featuring thioester groups within the linker region was designed and synthesized, thereby expanding the chemical linker toolbox for future ionizable lipid development. **Results**: Comprehensive in vivo structure–activity relationship studies led to the identification of CP-LC-1272 as a lead candidate that markedly enhances endosomal escape and exhibits superior in vivo biodegradability, attributed to the high acid-lability of thioester bonds. LNPs containing CP-LC-1272 maintained in vivo activity after six months of storage in lyophilized form and demonstrated superior in vivo efficiency compared to SM-102 in mRNA expression studies, as well as similar protein restoration in a tRNA delivery model targeting premature stop-codon mutations. **Conclusions**: The rapid biodegradability of these thioester-activated ionizable lipids (TAILs) suggests a reduced risk of accumulation, with the potential to enable safe repeated dosing or high-dosage RNA therapies, positioning TAILs as a versatile and safe platform for next-generation RNA therapeutics.

## 1. Introduction

RNA-based therapeutics have emerged as powerful strategies for the treatment and prevention of a broad spectrum of genetic disorders, infectious diseases, and cancer [1,2,3,4,5]. Their versatility lies in the ability to direct or modulate the synthesis of proteins and regulate cellular functions through multiple RNA modalities. Some of these include messenger RNA (mRNA), circular RNA (circRNA), transfer RNA (tRNA), and single guide RNA (sgRNA). Among these, mRNA has been the most clinically advanced, yet the growing interest in alternative RNA cargo types is rapidly expanding the therapeutic landscape.

Despite their transformative potential, RNA molecules are inherently unstable and prone to nuclease degradation, while their large size and negative charge hinder passive cellular uptake [6]. Overcoming these delivery challenges has been central to the progress of the field. Lipid nanoparticles (LNPs) have proven to be the most effective non-viral systems to date, shielding RNA cargo from degradation, enhancing cellular uptake, and facilitating endosomal escape. As a result, LNPs have enabled clinically viable RNA-based therapies [7,8], exemplified by FDA-approved drugs such as Onpattro, mRNA-1273, and BNT162b [9,10,11].

A typical LNP formulation consists of cholesterol, a PEGylated lipid, a phospholipid, and an ionizable lipid (IL) [12]. Among these, ionizable lipids are the most critical in protecting and transporting RNA cargo into the cytosol [13,14]. They offer the ability to remain neutral at physiological pH while becoming positively charged in the acidic endosome environment. This characteristic enables RNA condensation, efficient nanoparticle formation, and endosomal disruption through the promotion of non-bilayer structures [7,14,15]. Nevertheless, endosomal escape remains inefficient, with only a small fraction (~2–10%) of internalized RNA reaching the cytosol [16].

Following cellular uptake, LNPs are trafficked through the endocytic pathway, beginning with early endosomes, which progressively mature into late endosomes and ultimately fuse with lysosomes. Within lysosomes, foreign materials, including nucleic acids, are subject to enzymatic degradation [16,17,18]. Consequently, the timely release of the RNA cargo into the cytosol before lysosomal maturation is essential to preserve its functionality and achieve therapeutic efficacy. Their essential role in this mechanism has therefore motivated the development of next-generation ionizable lipids. These new designs are intended to enhance endosomal escape efficiency while also offering improved biodegradability, optimized pharmacokinetics, and superior safety profiles [19,20,21].

Beyond mRNA, other RNA cargos are increasingly recognized for their therapeutic potential. For instance, circRNAs offer extended stability [22,23,24]. sgRNAs enable CRISPR-mediated editing [25,26,27]. As for tRNAs, long appreciated as central components of protein synthesis, are emerging as promising candidates for restoring protein synthesis in genetic disorders mediated by premature STOP codon mutations [28]. The clinical exploration of these modalities requires delivery platforms that combine efficiency, safety, and biodegradability. Highly biodegradable ionizable lipids are particularly attractive in this regard, as they combine strong encapsulation and functional activity with favorable safety characteristics.

In this study, we designed and synthesized a novel library of ionizable lipids incorporating thioester linkages (TAILs) to enhance biodegradability while maintaining potent delivery activity. Through systematic structure–activity studies in vivo with mRNA encoding luciferase, we optimized the linker, hydrophobic tails, and head group chemistries. Lead candidates demonstrated significant improvements in both cellular uptake and endosomal escape, while also showing high biodegradability and favorable safety profiles. Stability testing further revealed that the optimized formulations retained activity after prolonged storage in both lyophilized and liquid forms. Finally, as proof of concept for alternative RNA cargos, we applied our best-performing TAILs to tRNA delivery, achieving high encapsulation and in vivo activity comparable to benchmark SM-102. Together, these results establish TAILs as a biodegradable and versatile platform for next-generation RNA therapeutics, including both mRNA and tRNA.

## 2. Materials and Methods

### 2.1. Chemicals

All chemical reagents were purchased from Sigma Aldrich (Burlington, MA, USA), Tokyo Chemical Industry (TCI, Tokyo, Japan), Fluorochem Ltd. (Hadfield, UK) and Ambeed (Arlington Heights, IL, USA). 1,2-dioleoyl-sn-glycero-3-phosphoethanolamine (DOPE) was purchased from Avanti Polar Lipids (Alabaster, AL, USA). 1,2-dimyristoyl-rac-glycero-3-methoxypolyethylene glycol-2000 (DMG-PEG 2000) was purchased from Cayman Chemicals (Ann Arbor, MI, USA). Cholesterol was purchased from Sigma Aldrich (Burlington, MA, USA). DLin-MC3-DMA (MC3) was purchased from Broadpharm (San Diego, CA, USA). 1-Octylnonyl 8-((2-hydroxyethyl) (6-oxo-6-(undecyloxy)hexyl)amino)octanoate (SM-102) ionizable lipid was purchased from BOC Sciences (Shirley, NY, USA). 6-((2-hexyldecanoyl)oxy)-N-(6-((2-hexyldecanoyl)oxy)hexyl)-N-(4-hydroxybutyl)hexan-1-aminium (ALC-0315) was purchased from Sigma Aldrich (Burlington, MA, USA).

### 2.2. mRNA Synthesis and tRNA Synthesis

mRNA was synthesized in vitro via transcription (IVT). The plasmid DNA (pDNA, GenScript, Piscataway, NJ, USA) encoding the target protein was linearized using BspQI (Hongene, Shanghai, China) according to the manufacturer’s instructions and purified with the Wizard^®^ SV Gel and PCR Clean-Up System (Promega, Madison, WI, USA).

Purified linear DNA served as the template for mRNA synthesis using T7 RNA polymerase. Transcription reactions were performed at 37 °C for 3 h in a mixture containing linear DNA (100 μg/mL), T7 RNA polymerase (5000 U/mL; Hongene, Shanghai, China), RNase inhibitor (1000 U/mL; Hongene, ON-039), inorganic pyrophosphatase (2 U/mL; Hongene, ON-025), ATP, GTP, CTP (each 5 μg/mL; Hongene, R1331/R2331/R3331), N^1^-methylpseudouridine (5 μg/mL; Hongene, R5-027), CleanCap^®^ AG (4 μg/mL; TriLink Biotechnologies, San Diego, CA, USA), and RNase-free double-distilled water.

The resulting mRNA was purified using an Oligo-dT resin column on an ÄKTA chromatography system (Cytiva, Marlborough, MA, USA), following the manufacturer’s protocol, yielding highly pure transcripts suitable for downstream applications.

tRNA was synthesized in vitro using a specifically designed dsDNA (GenScript, Piscataway, NJ, USA) template rather than a linearized plasmid. The IVT reaction was similar to that used for mRNA, with the following modifications: UTP (5 μg/mL; Hongene, R5331) was used instead of N^1^-methylpseudouridine, CleanCap^®^ AG was omitted, and the reaction was incubated for 5 h to optimize tRNA yield.

tRNA was subsequently purified using the Monarch^®^ Spin RNA Cleanup Kit (50 μg; NEB, Ipswich, MA, USA) according to the manufacturer’s protocol, effectively removing residual enzymes and unincorporated nucleotides.

### 2.3. Synthesis of Thioester Ionizable Lipids (TAILs)

All final thioester ionizable lipids were synthesized using a modular strategy involving three main steps: amine-functionalized thiolactone opening, thiol–acrylate Michael addition, and final acylation or esterification to introduce the hydrophobic tails (see Scheme S1 in Supplementary Materials). A list of all synthesized lipids and their respective purities (>95% by HPLC) is provided in Table 1.

### 2.4. Lipid Nanoparticle (LNP) Formulation

LNPs were prepared via microfluidics using an ethanol-based lipid solution containing ionizable lipid, DOPE (helper lipid), cholesterol, and DMG-PEG2000 (50:10:38.5:1.5 molar ratio). This was combined with an aqueous phase of mRNA or PTC-mRNA in 10 mM citrate buffer (pH 4) at a molar N/P ratio of 6/1. Mixing was performed using a NanoAssemblr^®^ IgniteTM device (Precision NanoSystems, Vancouver, BC, Canada) with a 3:1 flow-rate ratio (FRR) and 12 mL/min total flow rate (TFR). Post-formulation, LNPs were dialyzed overnight against a pH 8 Tris buffer with 15% sucrose. The final product was adjusted to 100 µg/mL mRNA, filtered (0.22 µm), and stored at 4 °C.

### 2.5. Lyophilized LNPs

After adjusting LNPs to a final concentration of 100 µg/mL RNA in Tris buffer (5 mM, pH 7.4) supplemented with 20% (*w*/*v*) maltose as a cryoprotectant, samples were aliquoted and lyophilized using a Virtis Genesis Pilot Freeze Dryer (SP, Warminster, PA, USA) following our previously described lyophilization process [29]. Briefly, the freeze-drying cycle included: (i) freezing at −50 °C for 5 h (ramp rate 1.6 °C/min), (ii) primary drying at −15 °C for 12 h under 180 mTorr (ramp rate 0.5 °C/min), and (iii) secondary drying at 30 °C for 7 h under 100 mTorr (ramp rate 0.5 °C/min). Upon completion of lyophilization, the vials were filled with pure nitrogen, sealed, and stored at 4 °C to assess stability. The lyophilized mRNA-LNP samples were maintained at 4 °C for one month and compared against non-lyophilized LNPs stored at −80 °C, which served as the control. For reconstitution, 300 µL of RNase-free water was added to each vial and gently mixed until a uniform, slightly whitish, clear suspension was achieved.

### 2.6. Formulation of tRNA LNPs

LNPs were prepared and stored according to the procedure described in the preceding section. The formulations were assembled using a molar N/P ratio of 3/1.

### 2.7. Characterization of LNPs

The average size, polydispersity index (PDI) and zeta potential of LNPs were determined using a Malvern Zetasizer Advance Lab Blue Label (Malvern Instruments Ltd., Malvern, UK) with a capillary cell (DTS1070) and diluting the sample (typically 1:100) in a 10 mM filtrated KCl solution. mRNA encapsulation efficiency was determined using the Quant-iT™ RiboGreen RNA assay (Thermo Fisher Scientific, Waltham, MA, USA) according to the manufacturer’s instructions. To measure the apparent pKa of LNPs, we performed a fluorescence-based assay using 6-(p-toluidinyl)naphthalene-2-sulfonic acid (TNS), following the method described by Kulkarni et al. [30]. Full characterization data are provided in Supplementary Information.

### 2.8. Cell Lines

Hela cells were obtained from the American Type Culture Collection (ATCC). Cells were cultured on Dulbecco’s Modified Eagle Medium (DMEM) with high glucose (Cytiva, SH30243), supplemented with 10% Fetal Bovine Serum (Sigma-Aldrich, St. Louis, MO, USA, F7524), 1% Penicillin-Streptomycin Solution (Gibco, Thermo Fisher Scientific, Waltham, MA, USA 15140122) and 2 mM Glutamax (Fisher Scientific, Thermo Fisher Scientific, Waltham, MA, USA, 35050038).

### 2.9. IncuCyte^®^ Assays

HeLa cells were seeded in Delta-treated 96-well plates at a density of 10,000 cell/well in complete DMEM and incubated at 37 °C and 5% CO_2_. After overnight incubation, culture medium was replaced, and mRNA-LNPs were added at a final concentration of 0.8 µg/mL (100 ng in 125 µL). Real-time analysis was performed using the IncuCyte^®^ SX5 Live-Cell Analysis System (Sartorius, Göttingen, Germany) at the Centro de Investigaciones Biomédicas de Aragón (Zaragoza, Spain; reference ES 50 297 0012 01). Acquisition times were 0, 1, 2, 4, 6, 8, 10, 12, 14, 16, 18, 20, 22, 24 h. The analysis was performed using the IncuCyte software (version 2024B).

### 2.10. Animals

All animal studies were performed in accordance with European and national regulations governing the protection of experimental animals. The experimental protocols were approved by the Ethics Committee for Animal Experiments at the University of Zaragoza (PI07/23).

Female BALB/cAnNRj mice, aged 8–10 weeks, were obtained from Janvier Labs. All animals were housed under specific pathogen-free conditions at the Centro de Investigaciones Biomédicas de Aragón. Upon arrival, the mice were acclimated for one week before the start of experiments. Environmental parameters were strictly controlled, with room temperatures maintained at 20–24 °C, relative humidity at 50–70%, light intensity at 60 lux, and a 12 h light/dark cycle. Food and water were available ad libitum throughout the study.

In all in vivo and ex vivo experiments, the experimental unit is the individual mouse. The sample size for each experiment was decided based on prior experience. No animals or data points were excluded from analysis. Animals were randomly assigned to treatment groups using a random number generator. Each animal was tagged with an individual number for identification purposes. Outcome assessors were blinded to group assignments during data analysis. Mice were monitored daily for signs of distress. No adverse events were observed.

### 2.11. HPLC Degradation

The appearance of metabolites A and B was monitored using HPLC-MS single ion recording with their theoretical *m*/*z*. In the case of CP-LC-1272, hydrolysis of the thioester bond yields Metabolite A, corresponding to the hydrophilic head group containing a dimethylaminoethylamide, and Metabolite B, consisting of the oleyl-derived fatty acid. In the case of SM-102, which contains an ester bond instead of a thioester, hydrolysis results in the formation of a tertiary amine–containing polar head group and linoleic acid as the hydrophobic tail. These degradation fragments are depicted in Figure 5A. Monitoring the release of these hydrolysis products by HPLC-MS enabled direct comparison of degradation profiles across lipid structures. Hence, 50 mM stock solutions of lipids NM2 (CP-LC-1284) and SM-102 were prepared in DMSO (TCI, >99% pure). Then, 20 µL of the stock solution was added to a vial containing 60 µL of pH = 5.2, 2.4 M aqueous buffer (sodium acetate, Sigma-Aldrich, St. Louis, MO, USA >99%) and 20 µL of DMSO (TCI, >99% pure). The measurements started immediately after the mixing, and then the vial was left stirring during the complete experiment, whilst HPLC injections were extracted every 42 min.

### 2.12. Pharmacokinetic Studies

BALB/c mice (*n* = 3, female), aged 8 weeks, were injected intravenously with 25 µg of Fluc mRNA per mouse encapsulated in LNPs (CP-LC-1272 or SM-102:DOPE:cholesterol:DMG-PEG2000 at molar ratios of 50:10:38.5:1.5). The LNPs were diluted in Tris buffer containing 15% sucrose and administered via tail vein injection in a final volume of 250 µL using a 27G syringe. Mice were euthanized by CO_2_ inhalation, and blood and liver samples were collected at various time points post-injection. Blood samples were allowed to clot and then centrifuged at 6500× *g* at 4 °C for 10 min to collect serum. For serum analysis, 100 µL of serum was diluted with 300 µL of a 1:1 mixture of isopropanol and ethanol, and an internal control was added. For liver analysis, 0.5 g of liver tissue was diluted in 1500 µL of the same isopropanol/ethanol mixture, and an internal control was added. The liver tissue was dissociated using a Gentle MACS Dissociator (Miltenyi, Biotec, Bergisch Gladbach, Germany). Dissociated liver and serum samples were sonicated for 15 min and centrifuged at 10,000× *g* for 15 min. The supernatants were collected and concentrated using a SpeedVac system (Eppendorf SE, Hamburg, Germany). Subsequently, supernatants were diluted with a 1:1 mixture of isopropanol and ethanol and analyzed against calibration standards. Chromatographic separation and quantification were accomplished with a liquid chromatography (LC)-MS system (Vanquish Core, Thermo Fisher Scientific, Waltham, MA, USA). Samples were injected and separated in an XBridge waters-BEH C8 column (Waters Corporation, Milford, MA, USA) equilibrated with 95% solvent A (H_2_O with 0.01% TFA) and 5% solvent B (Acetonitrile with 0.01% TFA). A simple quadrupole MS system (Waters Acquity QDa, Waters Corporation, Milford, MA, USA) operated in positive ion mode under SIR conditions was used for signal detection.

### 2.13. In Vitro S9 Assay

Mouse (CD-1) S9 Fractions were purchased from Thermofisher (MSS9PL), and degradation studies were performed following manufacturer instructions. Briefly, mouse liver S9 fractions (20 mg/mL) were thawed slowly on ice prior to use. Each reaction mixture was prepared containing 170 µL of TRIS buffer (200 mM solution including 2 mM magnesium chloride) at pH 7.4, 25 µL of LNPs and 5 µL of liver S9 fraction. The mixture was pre-incubated for 5 min at 37 °C in a water bath. Metabolic reaction was initiated by adding 10 µL of NADPH (20 mM), and incubations were carried out for up to 20 h at 37 °C under gentle agitation. Reactions were stopped by the addition of 1.5 mL of acetonitrile. Samples were vortexed and centrifuged at 8000 rcf for 10 min at 4 °C. The supernatant was collected and transferred to clean microtubes for LC–MS analysis. If immediate analysis was not possible, extracts were stored at −20 °C until analysis. Peak areas were normalized to the internal standard to correct variations in extraction efficiency and instrument response.

### 2.14. Hemolysis Assay

Erythrocytes were isolated from freshly collected heparinized human blood. Two milliliters of blood were washed three times with neutral PBS (pH 7.4) by centrifugation at 800× *g* for 5 min. The resulting erythrocyte pellet was then resuspended in 1 mL of either neutral PBS (pH 7.4) or acidic PBS (pH 6 or pH 5.5). The isolated erythrocytes were subsequently diluted 1:100 in PBS of the corresponding pH. For incubation, erythrocytes were exposed to LNPs at a final mRNA concentration of 2.5 μg/mL and maintained at 37 °C for 1 h. Following incubation, cells were centrifuged at 800× *g* for 5 min, and 60 μL of the supernatant was transferred to a 96-well plate. As a positive control for membrane disruption, 0.1% Triton X-100 was included. Absorbance was measured at 405 nm using a FLUOstar Omega plate reader (BMG Labtech, GmbH, Ortenberg, Germany).

### 2.15. In Vivo Safety Evaluation

Animals received an intravenous injection of luciferase-encoding mRNA (2.5 mg/kg) formulated in the specified LNPs as previously described. Blood samples were collected from the submandibular vein on day 2 post-inoculation, and serum was prepared for biochemical analysis. Samples were centrifuged at 8000 rcf for 10 min at 4 °C, and the resulting supernatants were collected. Biochemical assays were performed using the Cobas c-311 analyzer (Roche Diagnostics, GmbH, Mannheim, Germany) following the manufacturer’s protocol.

### 2.16. In Vivo Luminescence Assay of mRNA- and tRNA-LNPs

For intramuscular inoculation, mice were injected into the right thigh muscle using a 30G insulin syringe with 0.05 mg/kg of the indicated luciferase-encoding mRNA-LNPs diluted in Tris buffer containing 15% sucrose (final volume: 30 µL). For experiments involving tRNA-LNPs, a second intramuscular injection of 0.1 mg/kg of the specified tRNA-LNP formulation was administered into the same site 30 min after the initial PTC-mRNA -LNP injection. At 4 h post-final inoculation, mice were anesthetized by inhalation of 4% Isoflurane (IsoVet, Scientific Laboratory Supplies Ltd., Nottingham, UK) in an induction chamber and maintained under 1.5% isoflurane. D-luciferin (12507, Quimigen S.L., Madrid, Spain) diluted in PBS was administered intraperitoneally at 150 mg/kg. Luminescence images were acquired 20 min after luciferin injection using the IVIS Lumina XRMS Imaging System, following the manufacturer’s instructions.

### 2.17. Ex Vivo Luminescence Assay

Animals were administered 0.5 mg/kg of luciferase-encoding mRNA-LNPs via intravenous injection. The formulation was diluted in Tris buffer containing 15% sucrose to a final volume of 250 µL and delivered using a 27G needle. Four hours post-injection, mice were anesthetized in an inhalation chamber with 4% isoflurane (IsoVet) and maintained under anesthesia at 1.5% isoflurane throughout the procedure. D-luciferin (12507, Quimigen, Alverca do Ribatejo, Portugal), prepared in PBS, was administered intraperitoneally at a dose of 150 mg/kg. Twenty minutes following luciferin administration, luminescence imaging was performed using the IVIS Lumina XRMS Imaging System (Revvity Inc., Waltham, MA, USA) according to the manufacturer’s instructions.

### 2.18. Statical Analysis

Data are presented as mean ± SD or mean. One-way analysis of variance (ANOVA) followed by the Tukey test was applied for comparison using GraphPad Prism 10.0. Statistical significances are denoted in the Figures by asterisks, as follows: * *p*-value < 0.05, ** *p*-value < 0.01, *** *p*-value < 0.001, or **** *p*-value < 0.0001.

### 2.19. Protocol Registration

No formal protocol for the in vivo experiments described in this study was prospectively registered in a public repository. All procedures were, however, pre-defined and approved internally before study initiation and carried out in compliance with institutional and regulatory guidelines.

## 3. Results and Discussion

### 3.1. Design of Thioester-Activated Ionizable Lipids (TAILs)

**Figure 1:**
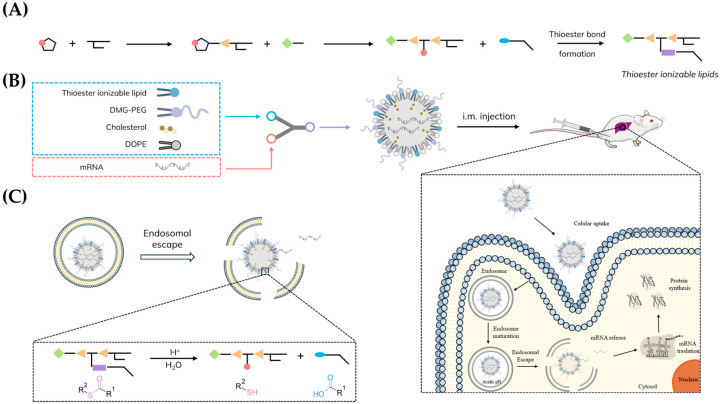
(**A**) Illustrative synthetic scheme for the synthesis of TAILs, highlighting the formation of the thioester bond. The code of colors used is as follows: Red circle: sulfur atom; orange triangle: linker substituent (amide or ester); green diamond: polar head; blue ellipse: carboxylic acid; purple rectangle: thioester bond. (**B**) Scheme of the process followed for the formulation of lipids into LNPs for mRNA delivery in vivo. LNPs were formulated by microfluidic mixing. (Right) Illustration of the delivery process of mRNAs into the cytosol via endosomal escape, enabling efficient protein translation, as mediated by LNPs. (**C**) Expanded scheme of the endosomal escape process, emphasizing the acid-triggered cleavage of the thioester bond within the ionizable lipid under endosomal pH conditions.

The architecture of ionizable lipids generally features a hydrophilic headgroup, two or more hydrophobic tails, and a molecular linker that joins these segments (Figure 1A). Modular design allows for significant structural diversity, with variations in the headgroup, linker, and tail regions influencing key aspects such as nanoparticle stability, delivery efficiency, pharmacokinetics, and biodegradability.

**Figure 2:**
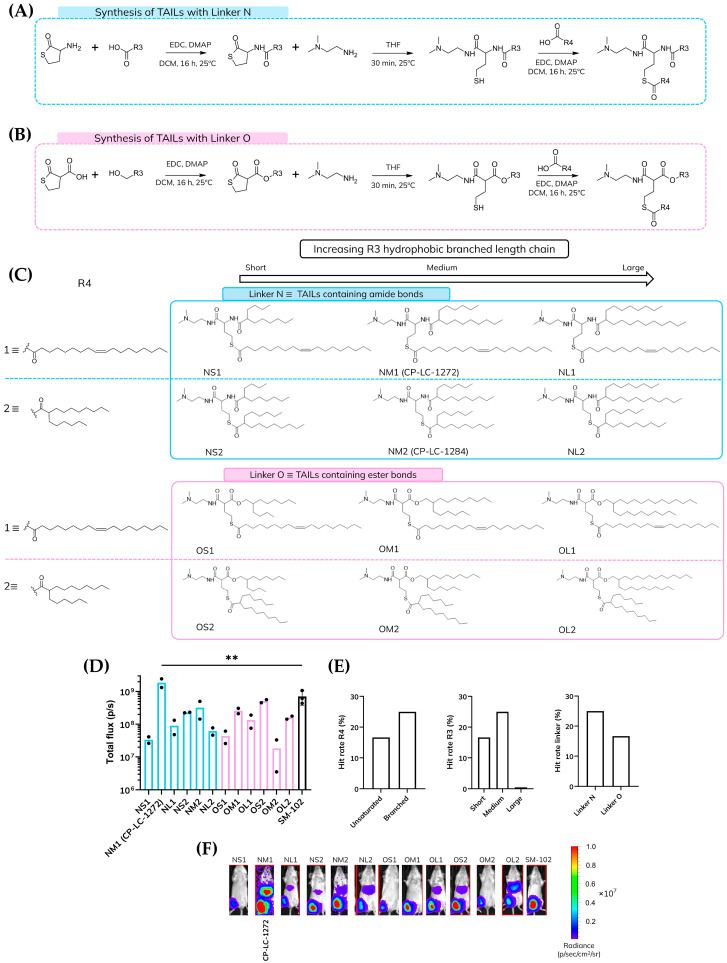
(**A**) Synthetic scheme describing TAILs synthetic steps with linker N. (**B**) Synthetic scheme describing TAILs synthetic steps with linker O. (**C**) Chemical structures with linker N or linker O modifying either R3 or R4 moiety. (**D**) In vivo Luc expression following administration of TAILs LNPs (*n* = 2 biologically independent samples) and control (*n* = 3). Data for TAILs are presented as mean values, while control data is shown as mean ± standard deviation (SD). ** *p* < 0.01 as determined by one-way ANOVA with Tukey post-test. Mice were i.m. injected with mRNA-Luc-loaded LNPs at an mRNA dose of 0.05 mg/kg. Luminescence imaging acquisition was performed at 4 h post-treatment, and total flux was quantified. Black dots within the bars represent individual measurements (**E**) Hit rate of TAIL LNPs grouped by modifications to R4, R3, and linker chemistry. Hit rate was defined as the proportion of lipid formulations within each structural category that achieved ≥25% of the protein expression observed with the SM-102 benchmark. Values are categorical and represent structural trends based on single screening outcomes rather than replicate measurements. (**F**) Representative luminescence images of each lipid in mice were acquired using the IVIS Lumina XRMS Imaging System at 4 h post-treatment.

The aim of this study was to investigate the incorporation of thioester functionalities into ionizable lipids as a strategy to obtain superior endosomal escape and superior biodegradability compared to typically used ester-containing lipids such as SM-102, while maintaining formulation stability (Figure 1B,C). To achieve this, we developed a library of ionizable lipids incorporating thioester groups, drawing inspiration from our previous work [31,32]. In these preceding studies, we first synthesized a thiolactone derivative via amide bond formation, followed by a one-pot, multicomponent reaction under mild conditions using the thiolactone derivative together with primary amines and acrylates. In the present library, however, the cycle of the thiolactone derivative was reacted with an amine. The resulting free thiol group was later reacted with a hydrophobic carboxylic acid to form a thioester functional group, yielding the final ionizable lipid (Figure 2A,B).

All thioester ionizable lipids used in this study were formulated into LNPs using microfluidic mixing, following the standard molar ratios established for the FDA-approved mRNA vaccine (mRNA-1273, Spikevax) [6] and encapsulating firefly luciferase or green fluorescence protein (GFP) mRNAs (Figure 1B).

To enable rigorous comparison across the synthesized TAILs and benchmark ionizable lipids such as SM-102 and MC3, all LNP formulations were prepared using the same molar ratios, nucleic acid cargo (mLuc or GFP mRNA), buffer systems, and microfluidic mixing protocol. Particle size, polydispersity index, ζ-potential, encapsulation efficiency, and apparent pKa were characterized for all formulations to confirm physicochemical comparability (see Supplementary Table S1). This uniformity in formulation and dosing conditions ensured that any observed differences in biological performance could be reliably attributed to differences in lipid structure.

### 3.2. Structure–Activity Optimization of Thioester Ionizable Lipids

To better understand how individual structural components influence the delivery performance of thioester ionizable lipids, we conducted a systematic optimization of their chemical architecture. Specifically, we examined how variations in the linker composition and hydrophobic tail design affect biodegradability and in vivo protein expression efficiency. The structural motifs explored in this library (variations in linker chemistry, hydrophobic tail length, and branching) were chosen based on insights from our previous work, where we screened nearly 100 ionizable lipids with diverse structural configurations [31,32]. This foundational SAR knowledge guided the rational design of the present TAIL library to ensure broad yet relevant chemical diversity and meaningful comparisons.

The final structures of the synthesized thioester-containing ionizable lipids incorporated a linker composed of either two amides and a thioester or an amide, an ester, and a thioester (Figure 2C). For clarity, herein we refer to these as linker N and linker O, respectively. Notably, by simply varying the chemical nature of the initial thiolactone ring, we were able to modulate one of the functional groups within the linker in a straightforward manner. This allowed us to demonstrate once again the versatility of the platform developed in our previous work [31].

In combination with linkers N and O, we further explored the influence of hydrophobic tail chemistry by modifying the length and nature of the R3 and R4 groups. Importantly, the R4 moiety is introduced during the thioester bond formation and is released upon hydrolysis, regenerating the original carboxylic acid. To evaluate the effect of this fragment on the performance of the lipid, we selected two distinct R4 chains: one branched and one unsaturated.

The branched R4 chain (substituent 2 in Figure 2C) was the same carboxylic acid used in lipid ALC-0315 (commercial lipid used in Pfizer/BioNTech’s vaccine) and in our previously identified top-performing lipid, CP-LC-0729, which exhibited no apparent toxicity [31]. In parallel, the unsaturated R4 chain (substituent 1 in Figure 2C) was synthesized from oleic acid, a natural compound which is considered safe and non-toxic at typical concentrations used in food, cosmetics, and pharmaceuticals [33,34].

For the R3 position, we selected hydrophobic chains of three different lengths (short, medium, and long) to systematically investigate the effect of tail length on lipid performance. The medium-length R3 chain corresponds to the same branched structure used for the R4 moiety. As the initial polar head group for this lipid library, we employed the optimized amine previously identified in our earlier screening studies [31]. This amine features a tertiary amine connected to a primary amine via two methylene units, and includes two methyl substituents on the tertiary amine, a configuration previously shown to yield high delivery performance.

To facilitate systematic analysis and comparison, we adopted a naming convention (XYZ) for the synthesized ionizable lipids, where each letter represents a distinct structural element: The first position (X) indicates the type of linker: “N” for nitrogen-based (amide) linkers and “O” for oxygen-based (ester) linkers. The second position (Y) denotes the length of the R3 hydrophobic chains: “S” for short, “M” for medium, and “L” for long chains. The third position (Z) refers to the nature of the R4 moiety: “1” for an unsaturated chain and “2” for a branched chain.

To assess the impact of structural modifications on delivery efficiency, we evaluated hit rates in vivo using LNPs encapsulating luciferase mRNA, with protein expression measured relative to a benchmark lipid. Ionizable lipids were classified as effective if they achieved at least 25% of the protein expression levels observed with the benchmark lipid SM-102 (Figure 2D). Among the variants tested, those containing nitrogen-based linkers (N linkers) with amide bonds exhibited the highest proportion of effective candidates (Figure 2E). This improved performance may be attributed to the hydrogen-bonding capacity of amide groups, which could enhance interactions between the ionizable lipid and the encapsulated mRNA, thereby facilitating improved in vivo expression [35,36]. Analysis of the R4 moiety revealed that branched chains resulted in a greater number of effective ionizable lipids. Nevertheless, the lipid NM1 (CP-LC-1272), which contains an unsaturated R4 chain, demonstrated the highest protein expression levels, surpassing all other candidates and the benchmark SM-102 (Figure 2D,F). In the case of the R3 moiety, where various branched chain lengths were compared, long-branched hydrophobic tails were found to be ineffective, exhibiting a 0% hit rate (Figure 2E). This observation suggests the existence of a maximum carbon chain length favorable for efficient protein expression. Accordingly, short and medium-length branched chains exhibited superior performance, with medium-length hydrophobic tails identified as the optimal configuration. These findings emphasize that careful structural tuning, particularly at the level of the linker and hydrophobic tails, is essential for achieving efficient protein expression. Guided by these observations, we identified the N linker, a medium-length tail, and an unsaturated R4 chain as the most favorable combination and used this configuration as the basis for subsequent studies of head group variation.

All LNP formulations exhibited favorable physicochemical characteristics, including high encapsulation efficiencies, appropriate particle sizes with low polydispersity, and suitable surface charges and apparent pKa values consistent with effective delivery performance. Detailed measurements are provided in Supplementary Table S1.

### 3.3. Impact of Polar Head on the Delivery Efficacy of Thioester Ionizable Lipids

Following the optimization of the linker and hydrophobic tail regions of the lipid structure, we further investigated the impact of various polar head groups while maintaining a constant chemical backbone.

**Figure 3:**
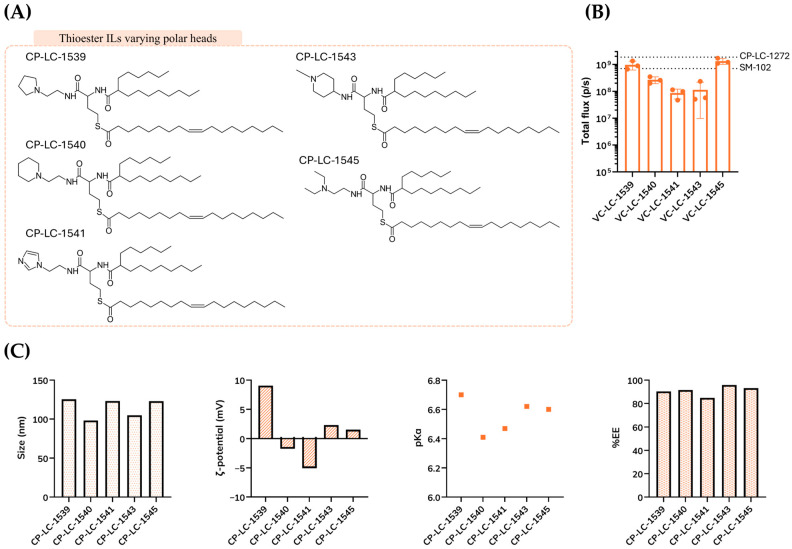
(**A**) Structures of ionizable lipids with different polar heads. (**B**) In vivo mLuc expression following administration of TAILs LNPs (*n* = 3 biologically independent samples). Data for TAILs are presented as mean values ± SD. Mice were i.m injected with mRNA-Luc-loaded LNPs at an mRNA dose of 0.05 mg/kg. Luminescence imaging acquisition was performed at 4 h post-treatment, and total flux was quantified. The dashed line on the y-axis represents the results of CP-LC-1272 and SM-102 LNPs. (**C**) Characterization of LNPs with TAILs. Each LNP was analyzed for hydrodynamic diameter, apparent pKa, ζ-potential and mRNA encapsulation efficiency (*n* = 3). Characterization data for LNPs are presented as mean values.

In line with this, we preserved a spacing of two methylene units between the amide bond and the tertiary amine within the polar head. Our earlier studies demonstrated that this specific spatial arrangement is critical for optimal delivery performance [31]. Consequently, we restricted our modifications to the nature of the tertiary amine incorporated in the polar head. This allowed us to isolate and evaluate the effects of head group structure on the overall lipid performance. Thus, we selected four lipids featuring cyclic polar head groups, guided by accumulating evidence in the literature highlighting the numerous advantages conferred by cyclic structures [37,38,39,40]. In addition, we incorporated an alternative polar head group featuring two ethylene group linkers on the tertiary amine, thereby expanding the structural diversity. This approach enabled the generation of five distinct thioester ionizable lipids (Figure 3A).

Interestingly, none of the newly tested lipids surpassed the in vivo potency of our previously developed lipid, CP-LC-1272. Notably, lipids CP-LC-1539 and CP-LC-1545 demonstrated higher protein expression levels than the benchmark lipid, SM-102, underscoring the superior performance of the selected linker and tail structures (Figure 3B). Regarding LNP characteristics, all formulations exhibited hydrodynamic diameters close to 100 nm and consistently high encapsulation efficiencies (>85%). The ζ-potentials ranged from −5.06 to 9.05 mV, and the apparent pKa values were approximately 6.5. Collectively, these results indicate that modifications to the polar head group did not significantly affect the physicochemical properties of the LNPs (Figure 3C).

For subsequent investigations, we selected the ionizable lipids CP-LC-1272, CP-LC-1539, and CP-LC-1545 based on their superior performance, positioning them as optimal candidates for advanced in vitro and in vivo evaluations.

### 3.4. Endosomal Escape and Cellular Uptake Assessment of Thioester Ionizable Lipids

Despite current efforts to discover systems to improve endosomal escape efficiency, endosomal escape remains a critical bottleneck for efficient mRNA release in LNP delivery systems [18,41]. Therefore, continued efforts to develop novel ionizable lipids with enhanced endosomal escape capabilities, alongside improved biodegradability, safety, and stability, are essential to advance the efficacy of mRNA therapeutics [19,41]. Thus, we hypothesized that ionizable lipids containing thioester moieties may improve endosomal escape due to their susceptibility to hydrolytic degradation in the endosome’s acidic pH environment. To test this hypothesis, cellular internalization and endosomal escape of fluorescently labeled lipid nanoparticles were assessed using an IncuCyte^®^ Live-Cell Analysis System in HeLa cells. To further analyze endosomal escape, mRNA expression was used as an indirect but functional readout. More specifically, mRNA translation served as a functional indicator of cytosolic delivery, allowing us to correlate LNP uptake with endosomal escape efficiency across formulations.

**Figure 4:**
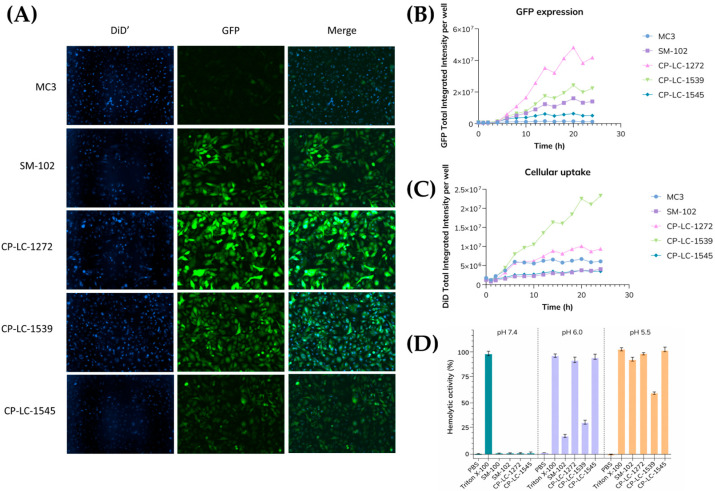
(**A**) HeLa cells were transfected with DiD’-labeled LNPs formulated with MC3, SM-102, CP-LC-1272, CP-LC-1539 or CP-LC-1545 encapsulating GFP mRNA at a dose of 0.8 µg/mL. Images were acquired at 14 h using an IncuCyte^®^ Live-Cell Analysis System. (**B**) Quantification of total GFP fluorescence intensity per well by time-lapse assay in HeLa cells to assess protein expression kinetics. (**C**) Corresponding total DiD fluorescence intensity per well measured at identical time points to evaluate LNP cellular uptake. (**D**) Hemolysis assays for CP-LC-1272, CP-LC-1539, CP-LC-1545, and SM-102 LNPs conducted at pH 7.4, 6.0, and 5.5. Red blood cells (RBCs) were incubated with each LNP formulation (1.25 µg mRNA/mL) for 1 h at 37 °C. 0.1% Triton X-100 and PBS were used as positive and negative controls, respectively. Data represent mean ± SD (*n* = 3 biologically independent samples).

From the initial screening of TAILs, CP-LC-1272, CP-LC-1539, and CP-LC-1545 were selected because they exhibit the highest levels of protein expression in vivo. These lipids differ only in their polar head groups and were compared with two established benchmarks for endosomal escape and intracellular trafficking, MC3 and SM-102 (Figure 4A–C). Cellular uptake was assessed using fluorescence-labeled lipid nanoparticles (Tables S2 and S3), with fluorescence intensity serving as a direct indicator of internalization. As shown in Figure 4C, CP-LC-1539 demonstrated the highest uptake, whereas SM-102, consistent with previous reports [19], showed poor uptake, a trend also observed for CP-LC-1545. CP-LC-1272 and MC3 exhibited intermediate uptake levels.

To quantitatively assess endosomal escape efficiency, the ratio of GFP expression was determined with respect to the measured fluorescence intensity of internalized LNPs (Figure S4). This approach allowed for the normalization of protein expression levels to cellular uptake, thereby isolating the contribution of endosomal escape from differences in nanoparticle internalization. The resulting endosomal escape ratio served as a comparative metric to evaluate the endosomal release performance of each ionizable lipid candidate (Table S4). The 20 h time point was selected for this analysis, as it corresponded to the peak of GFP expression (Figure 4B). At this point, CP-LC-1272 exhibited a markedly superior endosomal escape ratio compared to other lipids tested. Notably, CP-LC-1272’s endosomal escape ratio was 21.5-fold higher than MC3 and 1.1-fold greater than SM-102. Interestingly, while CP-LC-1539 and CP-LC-1545 showed lower endosomal escape ratios than CP-LC-1272 and SM-102, they still achieved 4.8- and 7.6-fold increases over MC3, respectively. These findings highlight the strong potential of the TAILs library to enhance endosomal escape. Nonetheless, the overall molecular architecture of the ionizable lipid clearly plays a decisive role in both cellular uptake and endosomal release, suggesting that these processes are closely governed by the chemical nature of the polar head group.

Apart from the real-time IncuCyte^®^ assays, the membrane-disruptive activity of the lipids was also evaluated as an indirect measure of endosomal escape, using hemolysis assays at physiological pH (7.4) and under acidic conditions (pH 6.0 and 5.5) [21,42]. CP-LC-1272 and CP-LC-1545 induced markedly stronger hemolysis than CP-LC-1539 and SM-102 at pH 6.0 (Figure 4D). However, at pH 5.5, closer to the environment of late endosomes, CP-LC-1539 displayed the lowest membrane-disruptive activity, consistent with its reduced endosomal escape ratio observed in IncuCyte^®^ assays (Table S4). All formulations showed negligible hemolytic activity at physiological pH, confirming their hemocompatibility.

These results corroborate live-cell imaging findings, reinforcing that CP-LC-1272 and CP-LC-1545 exhibit substantially higher endosomal escape capacities than CP-LC-1539 or SM-102. Importantly, assays performed at pH 5.5 provide a more physiologically relevant assessment of endosomal membrane disruption, as exemplified by the differential behavior of SM-102.

Collectively, these findings emphasize the importance of rational structural design in optimizing ionizable lipids for efficient intracellular delivery, with the polar head group and thioester functionality emerging as key determinants of endosomal escape efficiency. It is important to note, however, that in certain cell lines, protein expression may not directly reflect cytosolic mRNA availability, as it can also be influenced by differences in intracellular mRNA stability or cytosolic translation dynamics across formulations [17]. In the future, implementing direct detection tools, such as mRNA-specific reporters or fluorescent probes that signal upon cytosolic release, could provide more mechanistic insight and enable direct quantification of escape efficiency.

### 3.5. Pharmacokinetics and Biodegradability Assessment of TAILs

A widely used strategy to minimize or avoid potential toxic side effects has been the incorporation of ester bonds into ionizable lipid structures, given their susceptibility to enzymatic hydrolysis by esterases present in tissues and cells [10]. However, this degradation process can be relatively slow depending on the chemical structure of the lipid [19,43], as evidenced by the persistence of ionizable lipids for several days or even weeks [44,45]. Thioester bonds are weaker than ester bonds due to the less stable C(=O)–S linkage, which renders them more susceptible to nucleophilic attack by thiols, alcohols, or water [46]. Indeed, numerous examples exist of thioester bond degradation within cells during key biochemical processes (fatty acid β-oxidation, the Krebs cycle, and acetylcholine metabolism), where molecules containing thioester bonds, such as acetyl coenzyme A, are efficiently hydrolyzed. Based on these considerations, we hypothesized that incorporating thioester bonds into ionizable lipids would provide a superior alternative. This would allow for preserving efficiency while enhancing biodegradability.

This deliberate inclusion of a thioester functionality stems from its well-established chemical lability, which enables fast and controlled degradation in intracellular environments. Importantly, thioester hydrolysis yields a carboxylic acid and a thiol, both classes of compounds with precedents in physiological processes. In our case, the R4 chains released upon cleavage were either a branched alkyl acid present in ALC-0315 or oleic acid, a well-tolerated fatty acid widely used in pharmaceutical and food applications. These choices were made to mitigate concerns about metabolite safety while ensuring efficient elimination from the body.

**Figure 5:**
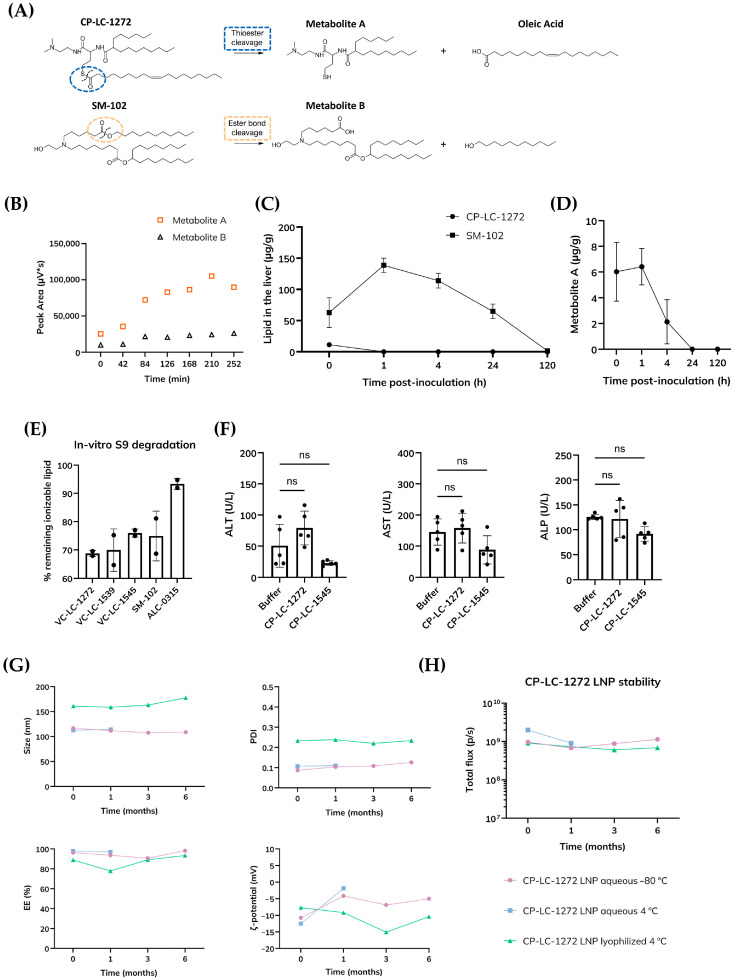
(**A**) (Top): Schematic representation of CP-LC-1272 degradation. Thioester bond cleavage yields Metabolite A and oleic acid, illustrating the generation of biocompatible fragments via acid-sensitive hydrolysis. (Bottom): Schematic representation of SM-102 degradation. Ester bond cleavage produces Metabolite B and 1-undecanol, which occurs at a slower rate under similar conditions. (**B**) Hydrolytic cleavage under mildly acidic conditions (pH 5.2), monitored by HPLC–MS detection of Metabolites A and B derived from NM2 (CP-LC-1284) and SM-102, respectively. (**C**) In vivo hepatic biodegradability of CP-LC-1272 and SM-102 LNPs evaluated at multiple time points (0, 1, 4, 24, and 120 h) post-injection, quantified by HPLC–MS. (**D**) Quantitative analysis of Metabolite A generated by thioester cleavage in liver samples collected at the same time points shown in (**C**). (**C**,**D**) Data represent mean ± SD (*n* = 3 biologically independent samples). Mice were i.m. injected with mRNA-Luc LNPs at a dose of 1.25 mg/kg mRNA. (**E**) In vitro degradation profile obtained using a liver S9 metabolic stability assay. Ionizable lipids were incubated for 20 h at 37 °C. Data represent mean ± SD (*n* = 2 biologically independent samples). (**F**) Serum levels of alanine aminotransferase (ALT), aspartate aminotransferase (AST), and alkaline phosphatase (ALP) following intravenous administration of mRNA-Luc LNPs (2.5 mg/kg mRNA; 50 µg RNA per mouse). Serum samples were collected 48 h post-injection for biochemical analysis. Data represent mean ± SD (*n* = 5 biologically independent samples). ns = not significant by one-way ANOVA with Tukey post-test. (**G**) Physicochemical characterization of CP-LC-1272 LNPs, including hydrodynamic diameter, polydispersity index (PDI), ζ-potential, and encapsulation efficiency, assessed at 0, 1, 3, and 6 months for samples stored at −80 °C and for lyophilized LNPs stored at 4 °C. Aqueous samples stored at 4 °C were evaluated at 0 and 1 month. (**H**) In vivo luciferase expression following administration of the CP-LC-1272 LNPs described in (**G**). Data represent mean values (*n* = 2 biologically independent samples). Mice were i.m. injected with mRNA-Luc LNPs (0.05 mg/kg mRNA). Luminescence images were acquired 4 h post-treatment, and total flux was quantified.

As an initial step to test this hypothesis, we assessed the hydrolytic degradation profiles of the thioester-containing lipid NM2 (CP-LC-1284) and the ester-containing control SM-102 under mildly acidic aqueous conditions (pH 5.2) using reverse-phase high-performance liquid chromatography (HPLC). CP-LC-1284 was chosen because its thioester is flanked by a branched alkyl substituent, a structural feature known in other contexts that slows ester hydrolysis (as observed for ALC-0315 compared to SM-102 [47]). Thus, CP-LC-1284 represents a stringent case for evaluating whether branching near the linkage could hinder thioester degradation. Hydrolysis of CP-LC-1284 released Metabolite A (the same degradation product observed for CP-LC-1272; Figure S5), while SM-102 cleavage generated Metabolite B (Figure 5A). Quantification of both metabolites by mass spectrometry revealed a clear difference in degradation kinetics: CP-LC-1284 underwent rapid hydrolysis, with Metabolite A levels substantially exceeding those of Metabolite B (Figure 5B). These results demonstrate that even in the presence of steric hindrance from a neighboring branch, thioester bonds undergo significantly faster acid-catalyzed hydrolysis than ester bonds under endosomal conditions. The accelerated degradation of CP-LC-1284 aligns with the intrinsic electrophilicity of the thioester carbonyl group, which facilitates nucleophilic attack by water molecules.

Next, we characterized the pharmacokinetic properties of our lead lipid, CP-LC-1272, using complementary in vitro and in vivo approaches. As observed with conventional LNPs, CP-LC-1272-based LNPs also preferentially accumulated in hepatic tissue, delivering and releasing their nucleic acid payload in the liver (Figure S6). Given this liver tropism, we employed the hepatic S9 fraction model to preliminarily assess metabolic stability. This in vitro system mimics liver metabolism and enables the evaluation of enzymatic degradation under well-controlled conditions [48]. CP-LC-1272, CP-LC-1539 and CP-LC-1545 LNPs were incubated with the S9 fraction alongside the clinically approved lipids SM-102 and ALC-0315 as controls. As shown in Figure 5E, CP-LC-1272, CP-LC-1539, and CP-LC-1545 exhibited markedly greater enzymatic degradability compared to ALC-0315, which displayed limited susceptibility to breakdown, consistent with its known metabolic stability [47].

Encouraged by these results, we next investigated the in vivo pharmacokinetics of CP-LC-1272, monitoring both the parent lipid and its primary metabolite (Metabolite A) in hepatic tissue over time (Figure 5C,D). CP-LC-1272 was rapidly degraded in the liver, and its quantification was not possible beyond one hour post-injection as it fell below the level of detection, whereas SM-102 persisted for up to 24 h, confirming the superior biodegradability of the thioester linkage (Figure 5C). In addition to hydrolysis, thioester bonds can undergo thiol-thioester exchange reactions [49], rendering them particularly susceptible to degradation in the presence of intracellular thiols such as glutathione (GSH) [46]. Given the high intracellular concentration of GSH, this mechanism likely contributes further to the enhanced biodegradability observed for TAILs.

To evaluate the safety profile of these lipids, in vivo toxicity studies were performed for CP-LC-1272 and CP-LC-1545 LNPs after an intravenous administration at a high dose (50 µg RNA per mouse, equivalent to 2.5 mg/kg). Liver function was assessed by quantifying serum biomarkers, including alkaline phosphatase (ALP), aspartate aminotransferase (AST), and alanine transaminase (ALT). No significant differences were detected between LNP-treated and PBS control groups, indicating that both formulations were well tolerated and did not induce hepatic injury (Figure 5F).

Overall, the high biodegradability and favorable safety profile of these thioester-based ionizable lipids constitute major advantages, minimizing the risk of long-term accumulation and associated toxicity. These features make TAILs particularly well suited for therapeutic applications that may require repeated or high-dose administration. However, it is important to acknowledge that while our data strongly suggest enhanced endosomal escape, they rely on indirect functional readouts, such as protein expression and hemolysis. In certain cell lines, protein expression may not directly reflect cytosolic mRNA availability, as it can also be influenced by differences in intracellular mRNA stability or translation kinetics. Therefore, future studies employing direct detection tools, such as cytosol-sensitive mRNA probes or split-reporter systems, will be valuable to validate and further elucidate the precise mechanisms by which thioester incorporation enhances intracellular delivery. Similarly, while the metabolic degradation profiles observed in vitro with liver S9 fractions, along with HPLC-MS confirmation of hydrolytic metabolites, suggest enhanced biodegradability of TAILs such as CP-LC-1272, these findings remain indirect. A full characterization of degradation pathways and clearance kinetics will require future in vivo pharmacokinetic studies, including long-term biodistribution and elimination analyses, to determine the extent to which these materials reduce accumulation relative to benchmark ionizable lipids.

### 3.6. Evaluation of CP-LC-1272 LNP Stability Under Refrigeration and Following Lyophilization

To evaluate whether CP-LC-1272 confers stability to LNPs despite its biodegradable nature, we conducted comprehensive storage studies under various conditions. LNPs were stored at 4 °C for one month, lyophilized and maintained for six months, or kept at −80 °C as a control. As shown in Figure 5H, all formulations preserved their in vivo efficiency across these conditions. Notably, our optimized freeze-drying process enabled the production of lyophilized LNPs that retained full biological activity for at least six months when stored at 4 °C (Section 2.5 for further details) [29,50].

Importantly, the stability of the thioester bond under physiological conditions was supported by LC-MS data showing minimal hydrolysis of CP-LC-1272 at pH 7.4. In contrast, enhanced degradation was observed under acidic conditions (pH 5.5–6.0), mimicking the endosomal environment (Figure 4D). These results indicate that the thioester remains stable during systemic circulation and storage, while still enabling rapid cleavage upon cellular uptake.

We attribute this stability to the pH-dependent hydrolysis behavior of the thioester bond. While thioesters undergo rapid cleavage under acidic conditions, their hydrolysis rate is markedly slower at physiological pH [49,51], allowing LNPs to remain intact during storage and systemic circulation while still enabling fast degradation in intracellular environments. This dual property of conditional lability makes the thioester linkage particularly advantageous for ionizable lipids used in RNA delivery.

Physicochemical characterization confirmed the robustness of the formulations, as particle size and polydispersity (PDI < 0.3) remained stable throughout the study, with no evidence of aggregation or structural alteration. A minor decrease in ζ-potential was observed in lyophilized CP-LC-1272 LNPs (from −7.79 mV to −15.09 mV), but this did not affect performance. All samples maintained high mRNA encapsulation efficiency over the six-month period (Figure 5G).

In summary, LNPs formulated with CP-LC-1272 combine long-term colloidal stability with rapid metabolic clearance upon administration, properties that are rarely achieved simultaneously in ionizable lipids. This balance between storage durability and controlled biodegradability highlights CP-LC-1272 as a promising candidate for safe, high-performance LNP formulations suitable for repeated or high-dose RNA therapeutic regimens.

Several classes of biodegradable ionizable lipids have been explored in recent years, including those featuring ester, carbonate, acetal, or ketal linkers [14,19,46,47]. These motifs have demonstrated improved metabolic clearance but often compromise formulation stability or delivery efficiency. In contrast, the thioester linkage introduced here is uniquely positioned to balance conditional lability with robust colloidal stability, as demonstrated by CP-LC-1272’s performance across long-term storage and in vivo models. Moreover, unlike some ester- or acetal-based systems that require additional excipients or stabilizers, our TAILs maintain their integrity without such modifications. This suggests that thioester incorporation may offer a new design strategy that overcomes typical stability–biodegradability trade-offs in lipid nanoparticle development.

### 3.7. Assessment of TAILs Potential in tRNA Delivery Systems for Suppression of Nonsense Mutations

As essential components of protein synthesis, tRNAs are emerging as promising therapeutic agents, particularly for genetic disorders [52] caused by mutations that disrupt mRNA translation. Nonsense mutations convert a sense codon, normally recognized by tRNA, into a premature termination codon (PTC), leading to truncated, nonfunctional proteins [28]. One strategy to overcome this involves the use of engineered tRNAs with modified anticodons capable of recognizing PTCs, thereby allowing translation to continue and restoring full-length protein expression. Despite the potential of this approach, the clinical development of tRNA-based therapeutics has been hindered by limited delivery efficiency and safety, and no such therapies have yet progressed to clinical trials. This highlights the urgent need for improved delivery platforms capable of enabling effective and safe tRNA administration.

**Figure 6:**
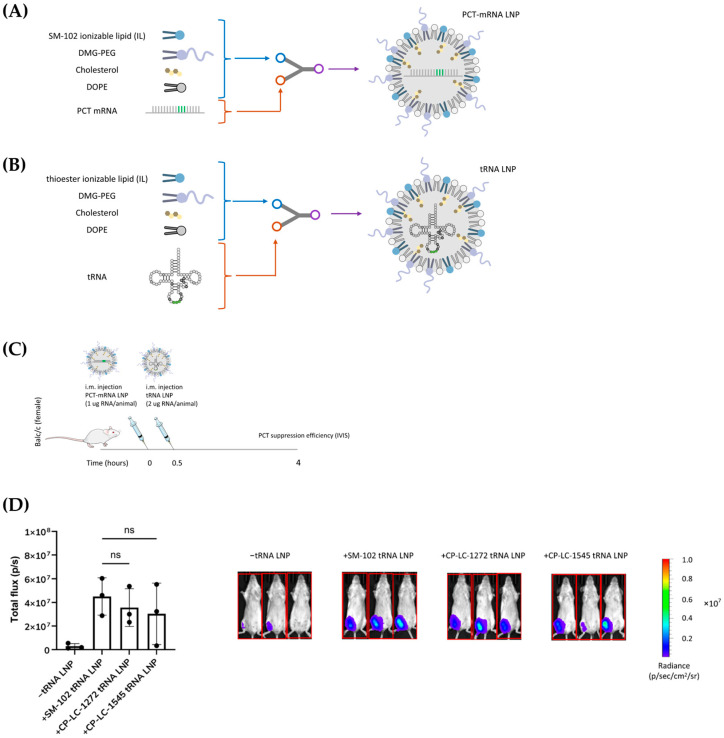
(**A**) Scheme of the process followed for the formulation of SM-102 LNP encoding PTC-mRNA LNP. LNPs were formulated by microfluidic mixing. (**B**) Scheme of the process followed for the formulation of LNPs encoding tRNA (tSA1T5). LNPs were formulated by microfluidic mixing. (**C**) Process followed for tRNA delivery for non-suppress mutation evaluation. (**D**) (Left) In vivo mLuc expression following i.m. administration of PTC-mRNA alone or combined with LNPs PTC-mRNA and tRNA (*n* = 3 biologically independent samples). Data are presented as mean values ± SD. ns non-significant as determined by one-way ANOVA with Tukey post-test. (Right) Bioluminescence images were obtained 4 h after treatment, and total flux was quantified to assess reporter expression. ns = not significant by one-way ANOVA with Tukey post-test.

To evaluate the potential of our TAILs for this application, we selected CP-LC-1272 and CP-LC-1545 to test their ability to deliver the tRNA tSA1T5, previously reported to restore protein translation in a nonsense mutation model [53]. In our study, mice were first administered LNPs formulated with SM-102 containing luciferase mRNA engineered with a premature termination codon (PTC-mRNA; Figure 6A,B). Thirty minutes later, LNPs encapsulating the engineered tRNA were injected. Bioluminescence images were acquired 4 h post-treatment to quantify protein expression restoration (Figure 6C).

Negative controls (mice not receiving tRNA LNPs) exhibited minimal luminescence, confirming efficient PTC suppression only in the presence of the tRNA (Figure 6D). In contrast, LNPs formulated with CP-LC-1272 or CP-LC-1545 achieved luminescence levels comparable to those obtained with SM-102, demonstrating that these TAILs enable efficient tRNA delivery and functional recovery of protein expression. Although further optimization of dosing and delivery parameters is needed, and additional validation across different mutation contexts or disease-relevant models would further strengthen the translational relevance of these findings, these results identify thioester-containing ionizable lipids as a promising platform for tRNA-based therapeutics.

## 4. Conclusions

We report a library of novel ionizable lipids that, to our knowledge, incorporate thioester functionality within the lipid scaffold for the first time. This design enabled a systematic structure–activity evaluation across linkers, polar head groups, and hydrophobic tails, yielding three lead candidates that outperformed the benchmark SM-102 in mRNA delivery. These leads combine thioester and amide linkages with an unsaturated oleyl-derived chain and a branched alkyl chain.

Among them, CP-LC-1272 emerged as the most effective in our studies, showing enhanced endosomal escape, consistent with the acid-labile nature of the thioester linkage, and rapid in vivo biodegradability in the liver. Importantly, LNPs formulated with CP-LC-1272 retained full activity after long-term storage both frozen and in the lyophilized state, indicating that enhanced biodegradability need not come at the expense of formulation stability.

Taken together, the balance of delivery performance, storage stability, and metabolic clearance observed for CP-LC-1272 supports the potential of thioester-containing ionizable lipids as promising candidates for future nucleic acid delivery applications. In a proof-of-concept tRNA model targeting nonsense mutations, CP-LC-1272 and CP-LC-1545 achieved protein restoration comparable to SM-102, suggesting that this chemistry may also be applicable beyond mRNA.

Overall, our results suggest that introducing thioesters into ionizable lipid frameworks provides a means to tune key delivery and biodegradation parameters. These findings provide a useful starting point for further exploration of thioester-based lipid design in other RNA delivery contexts and in repeat-dose studies.

## Figures and Tables

**Table 1:** Main data for the syntheses of thioester-containing lipids. For more information, see Supporting Information.

Lipid Name	Yield (%)	Purity (%)	[M + H]+ Theoretical	[M + H]+ Experimental
NS1	22	95	652.54	652.67
NS2	8.4	97	626.53	626.67
CP-LC-1272	31	97	708.61	708.80
NM2	26	96	682.59	682.78
NL1	5.1	98	764.67	764.87
NL2	11	98	738.65	738.86
OS1	17	95	667.54	667.77
OS2	16	96	641.53	641.71
OM1	21	97	723.61	723.84
OM2	44	99	697.59	697.80
OL1	13	97	835.73	835.94
OL2	64	96	809.72	809.89
CP-LC-1539	31	98	734.62	734.60
CP-LC-1540	25	97	748.64	748.60
CP-LC-1541	38	99	731.59	731.62
CP-LC-1543	11	98	734.62	734.60
CP-LC-1545	71	96	736.64	736.60

## Data Availability

All data supporting the findings of this study are included within the main article and its Supplementary Information Files.

## Supplementary Materials

The following supporting information can be downloaded at https://www.mdpi.com/article/10.3390/pharmaceutics18040472/s1.
