# Supplementary material for: Thioester-Containing Ionizable Lipids with Enhanced Endosomal Escape and Biodegradability for mRNA and tRNA Delivery
Source: Pharmaceutics. 2026 Apr 13;18(4):472. doi: 10.3390/pharmaceutics18040472 (PMC13118925; doi:10.3390/pharmaceutics18040472)
Supplement: Supplementary file 1 [file pharmaceutics-18-00472-s001.zip › pharmaceutics-4206928-supplementary.pdf]

Supplementary Information for

Thioester-containing ionizable lipids with enhanced endosomal escape and biodegradability for mRNA and tRNA delivery

Álvaro Peña[1], Esther Broset[1], Enrique Lucia[1], Laura García-Latorre[1], Víctor Navarro[1], Carlos Matute[1], Ana Gallego-Lleyda[1], Teresa Alejo[1], Eduardo Romanos,[2] Alba García,[2] Juan Martínez-Oliván[1,]* & Javier Giménez-Warren[1,*].

[1]Certest Pharma, Certest Biotec S. L., 50840, San Mateo de Gállego (Zaragoza), Spain.

[2]Medical Imaging and Phenotyping Core Facility, Aragon Health Sciences Institute (IACS), 50009 Zaragoza, Spain;

*Corresponding authors: Juan Martínez-Oliván and Javier Giménez-Warren (ORCID No.: 0000-0002-1747-6393)

Email addresses: A.P.: apena@certest.es; E.B.: ebroset@certest.es; E.L.: elucia@certest.es; L.G.-L.: lgarcia@certest.es; V.N.: vnavarro@certest.es; C.M.: cmatute@certest.es; A.G.-L.: agallego@certest.es; T.A.: talejo@certest.es; E.R.: eromanos.iacs@aragon.es; A.G.: agarciagil.iacs@aragon.es; J.M.-O.: jmartinez@certest.es; J.G.-W.: jgimenez@certest.es

Procedure For Thioester-Containing Ionizable Lipid Synthesis

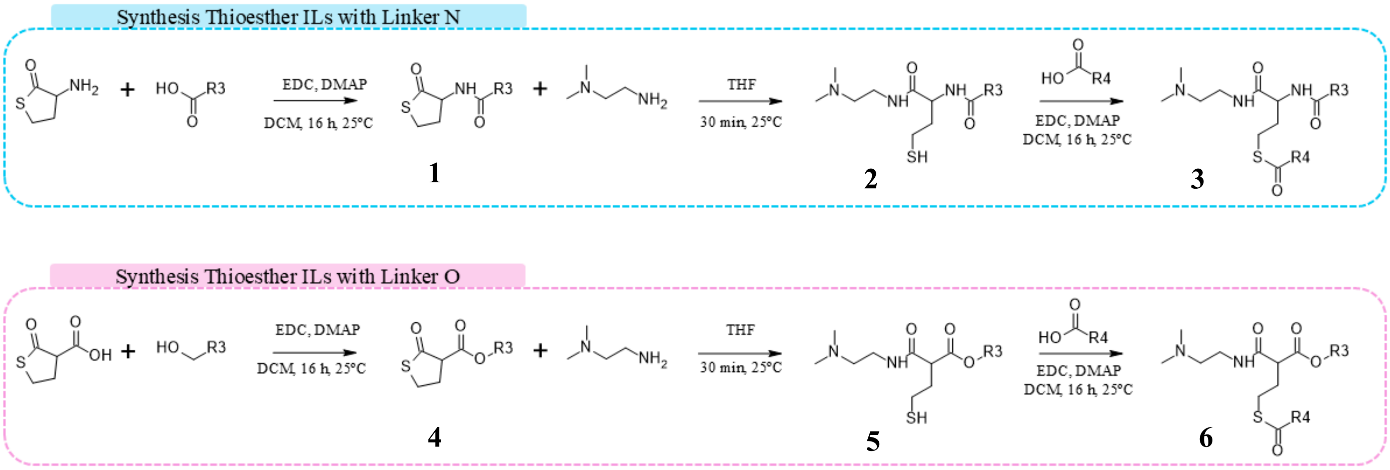

Scheme S1

General Synthesis Thioester ILs with linker N

Synthesis of **1**: DL-homocysteine thiolactone hydrochloride (270.4 mg, 1.76 mmol; Sigma Aldrich (Burligton, Massachusetts, USA, 99% purity) was dissolved in 5 mL of anhydrous dichloromethane (TCI, Tokyo, Japan; 99% purity) at room temperature. Then triethylamine (1620 µL, 1.8 mmol, Sigma Aldrich (Burligton, Massachusetts, USA, 99% purity) was added followed by EDC hydrochloride (249.2 mg, 1.3 mmol, Sigma Aldrich (Burligton, Massachusetts, USA, 98% purity), 4-(dimethylamino)pyridine (24.4 mg, 0.2 mmol, Sigma Aldrich (Burligton, Massachusetts, USA, 99% purity) and the respective hydrophobic carboxylic acid (1 mmol, Ambeed (Arligton Heights, Illinois, USA, >95%). The reaction mixture was stirred at room temperature overnight under an argon atmosphere. Then, the reaction crude was washed two times using distilled water (2 x 10 mL) and finally with saturated brine (10 mL). The organic layer was dried with anhydrous MgSO4, filtered, and evaporated under reduced pressure. The resulting residue was purified by flash chromatography (gradient of hexane/ethyl acetate: 100/0 to 0/100) to afford **1**.

Synthesis of **2**: The previous product 1 (0.7 mmol) was dissolved in anhydrous tetrahydrofuran (1.5 mL, TCI, Tokyo, Japan; 98% purity) and was added to a solution of N,N-Dimethylethylenediamine (756 uL, 7.03 mmol, Sigma Aldrich (Burligton, Massachusetts, USA, 99% purity) in anhydrous tetrahydrofuran (1.0 mL) under argon atmosphere. The solution was then stirred at room temperature for 30 minutes, after which the solvent was evaporated under reduced pressure. The dry mixture was redissolved in ethyl acetate (20 mL, TCI, Tokyo, Japan; 99% purity) and washed with a solution of 0.1 M HCl (aq) (20 mL, 3 consecutive washes), then distilled water (20 mL, 3 consecutive washes) and finally with saturated brine (NaCl:water prepared by adding 35 g of NaCl in 100 mL of water; 20 mL, 3 consecutive washes;). The combined organic fractions were dried with MgSO4 (anh) and subsequently evaporated under reduced pressure to yield **2** (quantitative yield).

Synthesis of final TILs **3**: The product from the previous step (**2**, 177.5 mg, 0.4 mmol), EDC hydrochloride (101.7 mg, 0.52 mmol), 4-(dimethylamino)pyridine (9.8 mg, 0.08 mmol) and oleic acid (153.8 µL, 0.48 mmol, Sigma Aldrich (Burligton, Massachusetts, USA, 98% purity) were dissolved in 2.5 mL of anhydrous dichloromethane at room temperature. The reaction mixture was stirred at room temperature overnight under argon atmosphere. Then, the reaction crude was redissolved in 40 mL of dichloromethane and washed two times using distilled water (2 x 10 mL) and finally with saturated brine (10 mL). The organic layer was dried with anhydrous MgSO4,

filtered, and evaporated under reduced pressure. The resulting residue was purified by flash chromatography (gradient of dichloromethane/eluent A: 100/0 to 0/100). After removing the solvent under reduced pressure, final ionizable lipid **3** is obtained.

*Eluent A: dichloromethane/methanol/ammonium hydroxide (80:20:1)

General Synthesis Thioester ILs with linker O

Synthesis of **4**: 2-Oxotetrahydrothiophene-3-carboxylic acid (175 mg, 1.2 mmol, BOC Sciences (NY, USA, 97% purity) was dissolved in 5 mL of anhydrous dichloromethane at room temperature. Then the respective hydrophobic alcohol (1.0 mmol, Ambeed (Arligton Heights, Illinois, USA, >95%) was added followed by EDC hydrochloride (254 mg, 1.3 mmol, Sigma Aldrich (Burligton, Massachusetts, USA, 98% purity) and 4-(dimethylamino)pyridine (25 mg, 0.2 mmol, Sigma Aldrich (Burligton, Massachusetts, USA, 99% purity). The reaction mixture was stirred at room temperature overnight under an argon atmosphere. After that, the reaction mixture was first washed twice with water and finally with brine. The organic layer was dried with anhydrous MgSO4, filtered, and evaporated under reduced pressure. The resulting residue was purified by flash chromatography (gradient of hexane/dichloromethane: 100/0 to 0/100) to afford the product **4**.

Synthesis of **5**: An identical procedure to that described for the preparation of compound 2 was applied, substituting compound 4 as the starting material.

Synthesis of **6**: The same experimental conditions described for compound 3 was repeated, with compound 4 as the initial product.

2-butyl-N-(2-oxotetrahydrothiophen-3-yl)octanamide

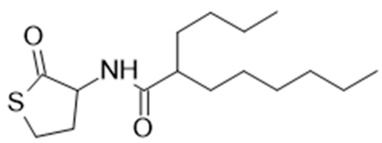

Yield 78%. The pure compound (93%) was characterized by mass spectroscopy. Theoretical $[M+H]^+$=300.20, experimental $[M+H]^+$=300.30

N-(2-hexyldecyl)-2-oxotetrahydrothiophene-3-carboxamide.

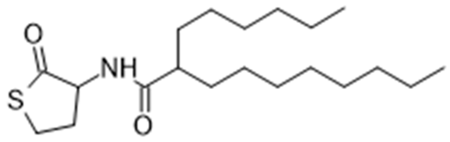

Yield 67%. The pure compound (91%) was characterized by mass spectroscopy. Theoretical $[M+H]^+$=370.28, experimental $[M+H]^+$=370.46

2-octyl-N-(2-oxotetrahydrothiophen-3-yl)dodecanamide

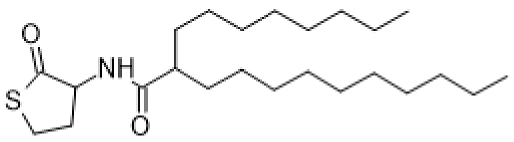

Yield 58%. The pure compound (94%) was characterized by mass spectroscopy. Theoretical $[M+H]^+$=412.32, experimental $[M+H]^+$=412.50

2-butyloctyl 2-oxotetrahydrothiophene-3-carboxylate.

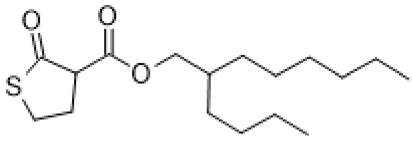

Yield 74%. The pure compound (89%) was characterized by mass spectroscopy. Theoretical $[M+H]^+$=315.19, experimental $[M+H]^+$=315.35

2-hexyldecyl 2-oxotetrahydrothiophene-3-carboxylate.

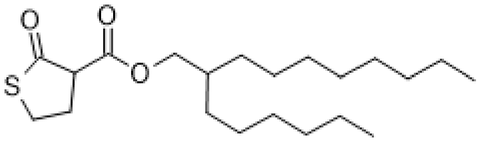

Yield 84%. The pure compound (93%) was characterized by mass spectroscopy. Theoretical $[M+H]^+$=371.26, experimental $[M+H]^+$=371.27

2-decyltetradecyl 2-oxotetrahydrothiophene-3-carboxylate

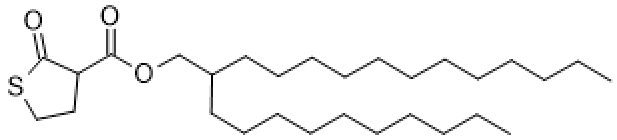

Yield 63%. The pure compound (91%) was characterized by mass spectroscopy. Theoretical $[M+H]^+$=483.39, experimental $[M+H]^+$=483.45

2-butyl-N-(2-oxotetrahydrothiophen-3-yl)octanamide

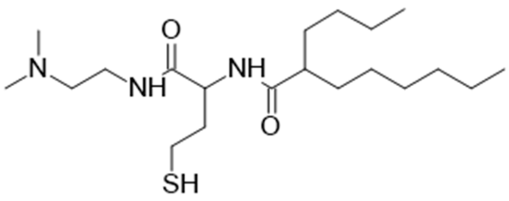

Quantitative yield. The pure compound (92%) was characterized by mass spectroscopy. Theoretical $[M+H]^+$ =300.20, experimental $[M+H]^+$ =300.30

N-(1-((2-(dimethylamino)ethyl)amino)-4-mercapto-1-oxobutan-2-yl)-2-hexyldecanamide

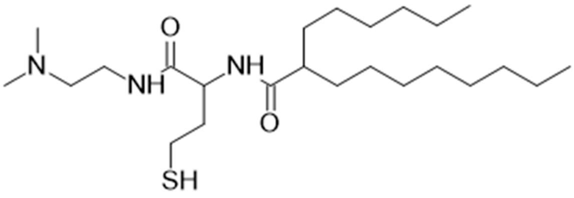

Quantitative yield. The pure compound (87%) was characterized by mass spectroscopy. Theoretical $[M+H]^+$ =444.36, experimental $[M+H]^+$ =444.35

N-(1-((2-(dimethylamino)ethyl)amino)-4-mercapto-1-oxobutan-2-yl)-2-octyldodecanamide

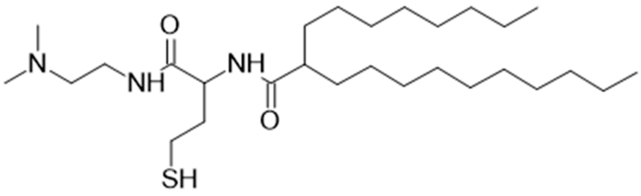

Quantitative yield. The pure compound (91%) was characterized by mass spectroscopy. Theoretical $[M+H]^+$ =500.43, experimental $[M+H]^+$ =500.56

2-butyloctyl 2-((2-(dimethylamino)ethyl)carbamoyl)-4-mercaptobutanoate

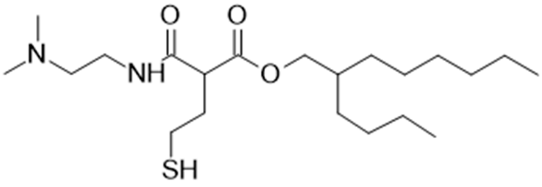

Quantitative yield. The pure compound (92%) was characterized by mass spectroscopy. Theoretical $[M+H]^+$ =403.39, experimental $[M+H]^+$ =403.26

2-hexyldecyl 2-((2-(dimethylamino)ethyl)carbamoyl)-4-mercaptobutanoate

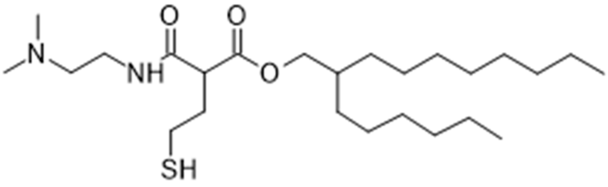

Quantitative yield. The pure compound (91%) was characterized by mass spectroscopy. Theoretical $[M+H]^+$ =459.36, experimental $[M+H]^+$ =459.35

2-decyltetradecyl 2-((2-(dimethylamino)ethyl)carbamoyl)-4-mercaptobutanoate

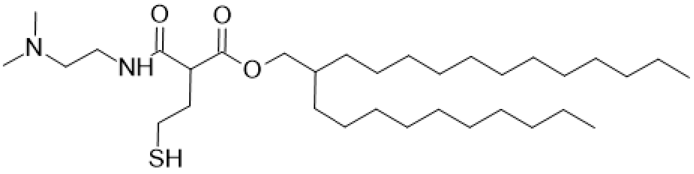

Quantitative yield. The pure compound (99%) was characterized by mass spectroscopy. Theoretical $[M+H]^+$ =571.48, experimental $[M+H]^+$ =571.58

NS1 (S-(3-(2-butyloctanamido)-4-((2-(dimethylamino)ethyl)amino)-4-oxobutyl) (Z)-octadec-9-enethioate): Yield 22 %. The pure compound (95%) was characterized by mass spectroscopy. Theoretical $[M+H]^+$ =652.54, experimental $[M+H]^+$ =652.67

NS2 (S-(3-(2-butyloctanamido)-4-((2-(dimethylamino)ethyl)amino)-4-oxobutyl) 2-hexyldecanethioate): Yield 8.4 %. The pure compound (97%) was characterized by mass spectroscopy. Theoretical $[M+H]^+$ =626.53, experimental $[M+H]^+$ =626.67

CP-LC-1272 (NM1) (S-(4-((2-(dimethylamino)ethyl)amino)-3-(2-hexyldecanamido)-4-oxobutyl) (Z)-octadec-9-enethioate): Yield 31%. The pure compound (97%) was characterized by mass spectroscopy. Theoretical $[M+H]^+$ =708.61, experimental $[M+H]^+$ =708.8. ^{1}H NMR (500 MHz, CDCl3) δ: 6.73 (t, J = 5.1 Hz, 1H), 6.35 (d, J = 7.8 Hz, 1H), 5.34 (m, 2H), 4.45 (m, 1H), 3.33 (dt, J = 5.9, 5.9 Hz, 2H, 2H), 2.96 (m, 1H), 2.76 (m, 1H), 2.54 (t, J=7.4 Hz, 2H), 2.42 (m, 2H), 2.23 (s, 6H), 2.14 – 2.04 (m, 2H), 2.00 (m, 4H), 1.89 (m, 1H), 1.68 – 1.54 (m, 4H), 1.46 – 1.37 (m, 2H), 1.36 – 1.17 (m, 40H), 0.90 – 0.83 (m, 9H).

NM2 (S-(4-((2-(dimethylamino)ethyl)amino)-3-(2-hexyldecanamido)-4-oxobutyl) 2-hexyldecanethioate): Yield 26%. The pure compound (96%) was characterized by mass spectroscopy. Theoretical $[M+H]^+$ =682.59, experimental $[M+H]^+$ =682.78

NL1 (S-(4-((2-(dimethylamino)ethyl)amino)-3-(2-octyldodecanamido)-4-oxobutyl)

(Z)-octadec-9-enethioate): Yield 5.1 %. The pure compound (98%) was characterized by mass spectroscopy. Theoretical $[M+H]^+$ =764.67, experimental $[M+H]^+$ =764.87

NL2 (S-(4-((2-(dimethylamino)ethyl)amino)-3-(2-octyldodecanamido)-4-oxobutyl) 2-

hexyldecanethioate): Yield 11%. The pure compound (98%) was characterized by mass spectroscopy. Theoretical $[M+H]^+$ =738.65, experimental $[M+H]^+$ =738.86

OS1 (2-butyloctyl 2-((2-(dimethylamino)ethyl)carbamoyl)-4-(oleoylthio)butanoate): Yield 17%. The pure compound (95%) was characterized by mass spectroscopy. Theoretical $[M+H]^+$ =667.54, experimental $[M+H]^+$ =667.77

OS2 (2-butyloctyl 2-((2-(dimethylamino)ethyl)carbamoyl)-4-((2-hexyldecanoyl)thio)butanoate): Yield 16%. The pure compound (96%) was characterized by mass spectroscopy. Theoretical $[M+H]^+$ =641.53, experimental $[M+H]^+$ =641.71

OM1 (2-hexyldecyl 2-((2-(dimethylamino)ethyl)carbamoyl)-4-(oleoylthio)butanoate): Yield 21%. The pure compound (97%) was characterized by mass spectroscopy. Theoretical $[M+H]^+$ =723.61, experimental $[M+H]^+$ =723.84

OM2 (2-hexyldecyl 2-((2-(dimethylamino)ethyl)carbamoyl)-4-((2-hexyldecanoyl)thio)butanoate): Yield 44%. The pure compound (99%) was characterized by mass spectroscopy. Theoretical $[M+H]^+$ =697.59, experimental $[M+H]^+$ =697.80

OL1 (2-decyltetradecyl 2-((2-(dimethylamino)ethyl)carbamoyl)-4-(oleoylthio)butanoate): Yield 13%. The pure compound (97%) was characterized by mass spectroscopy. Theoretical $[M+H]^+$ =835.73, experimental $[M+H]^+$ =835.94

OL2 (2-decyltetradecyl 2-((2-(dimethylamino)ethyl)carbamoyl)-4-((2-hexyldecanoyl)thio)butanoate): Yield 64%. The pure compound (96%) was characterized by mass spectroscopy. Theoretical $[M+H]^+$ =809.72, experimental $[M+H]^+$ =809.89

CP-LC-1539 (S-(3-(2-hexyldecanamido)-4-oxo-4-((2-(pyrrolidin-1-yl)ethyl)amino)butyl) (Z)-octadec-9-enethioate): Yield 31%. The pure compound (98%) was characterized by mass spectroscopy. Theoretical $[M+H]^+$ =734.62, experimental $[M+H]^+$ =734.60. ^{1}H NMR (500 MHz, $CDCl_3$) δ: 7.06 (s, 1H), 6.58 (d, J = 7.9 Hz, 1H), 5.35 (m, 2H), 4.49 (m, 1H), 3.50 (m, 1H), 3.37 (m, 1H), 2.95 (m, 1H), 2.81 – 2.67 (m, 7H), 2.53 (t, J = 7.6 Hz, 2H), 2.21 – 2.06 (m, 2H), 2.06 – 1.96 (m, 4H), 1.96 – 1.89 (m, 1H), 1.86 (m, 4H), 1.69 – 1.54 (m, 4H), 1.41 (m, 2H), 1.32 – 1.20 (m, 40H), 0.86 (m, 9H).

CP-LC-1540 (S-(3-(2-hexyldecanamido)-4-oxo-4-((2-(piperidin-1-yl)ethyl)amino)butyl) (Z)-octadec-9-enethioate): Yield 25%. The pure compound (97%)

was characterized by mass spectroscopy. Theoretical $[M+H]^+$ =748.64, experimental $[M+H]^+$ =748.60

CP-LC-1541 (S-(4-((2-(1H-imidazol-1-yl)ethyl)amino)-3-(2-hexyldecanamido)-4-oxobutyl) (Z)-octadec-9-enethioate): Yield 38%. The pure compound (99%) was characterized by mass spectroscopy. Theoretical $[M+H]^+$ =731.59, experimental $[M+H]^+$ =731.62

CP-LC-1543 (S-(3-(2-hexyldecanamido)-4-((1-methylpiperidin-4-yl)amino)-4-

oxobutyl) (Z)-octadec-9-enethioate): Yield 11%. The pure compound (98%) was characterized by mass spectroscopy. Theoretical $[M+H]^+$ =734.62, experimental $[M+H]^+$ =734.60

CP-LC-1545 (S-(4-((2-(diethylamino)ethyl)amino)-3-(2-hexyldecanamido)-4-oxobutyl) (Z)-octadec-9-enethioate): Yield 71%. The pure compound (96%) was characterized by mass spectroscopy. Theoretical $[M+H]^+$ =736.64, experimental $[M+H]^+$ =736.60. ^1H NMR (500 MHz, CDCl3) δ: 6.85 (m, 1H), 6.40 (d, J = 7.7 Hz, 1H), 5.39 – 5.29 (m, 2H), 4.46 (m, 1H), 3.33 (dt, J = 5.9, 5.9 Hz, 2H), 2.95 (m, 1H), 2.77 (m, 1H), 2.63 – 2.49 (m, 8H), 2.12 – 2.05 (m, 2H), 2.03 – 1.97 (m, 4H), 1.93 – 1.84 (m, 1H), 1.67 – 1.54 (m, 4H), 1.47 – 1.36 (m, 2H), 1.34 – 1.19 (m, 41H), 1.03 (t, J = 7.1 Hz, 6H), 0.86 (m, 9H).

NMR SPECTRA

A)

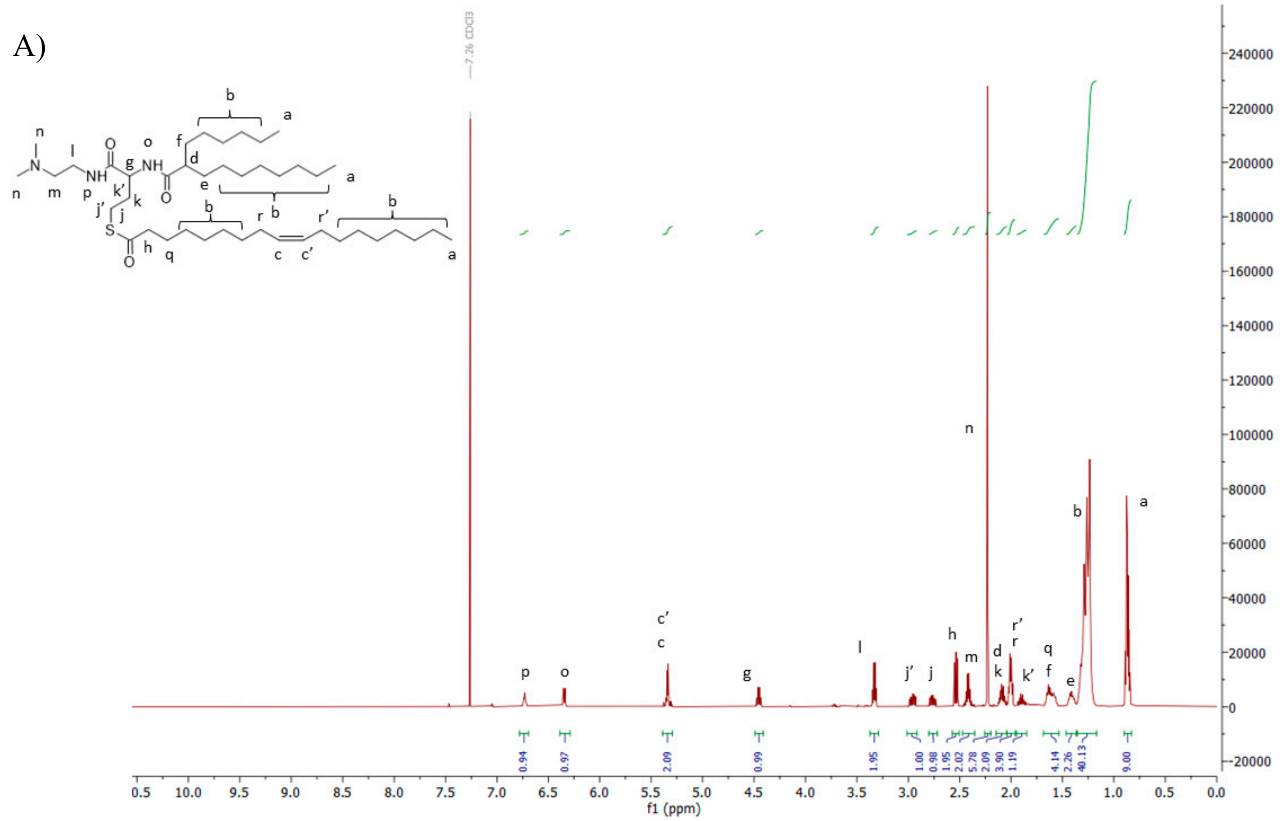

B)

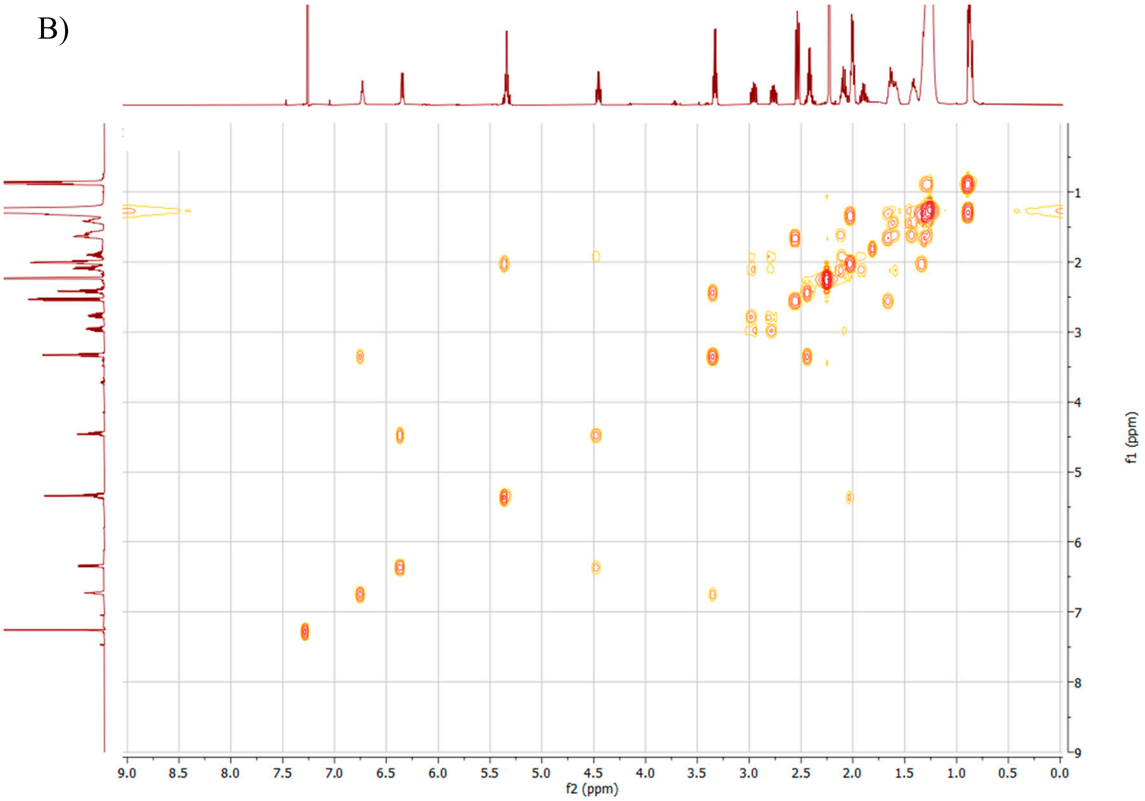

Figure S1. (A) ^{1}H-NMR spectra ($CDCl_3$, 500 MHz) of ionizable lipid CP-LC-1272 (NM1) with peak assignment. **(B)** 2D COSY spectrum of ionizable lipid CP-LC-1272 (NM1).

A)

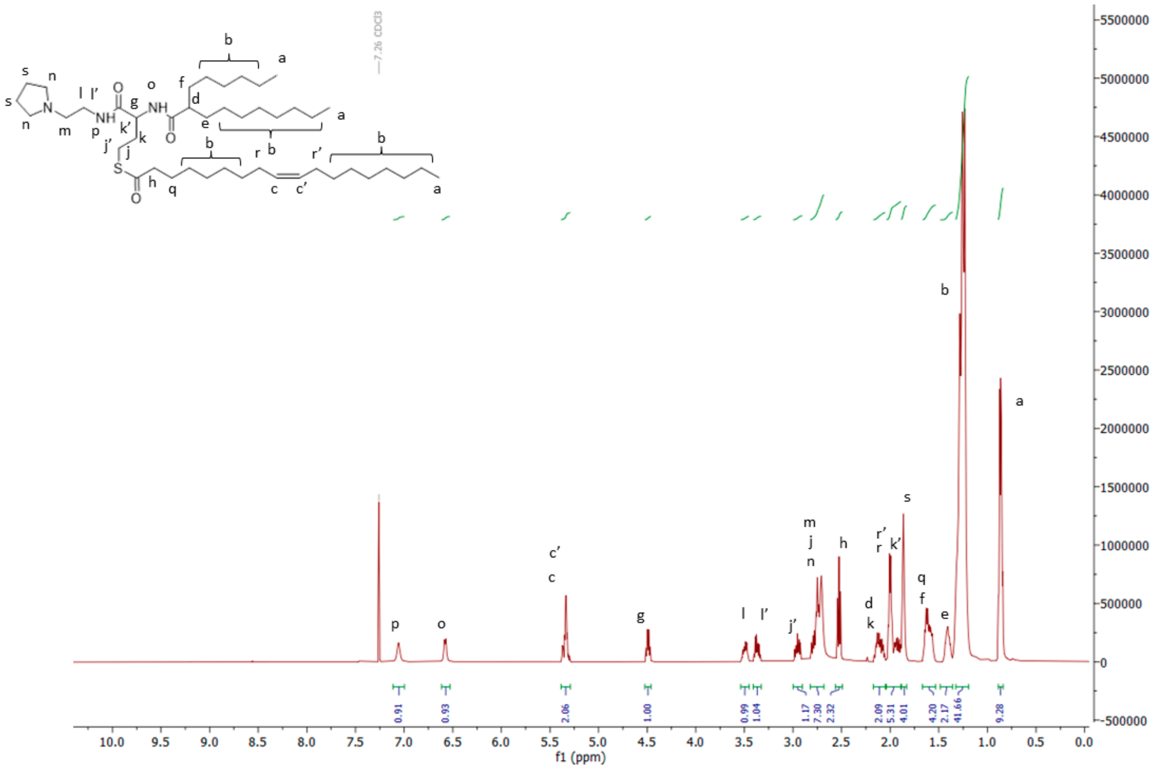

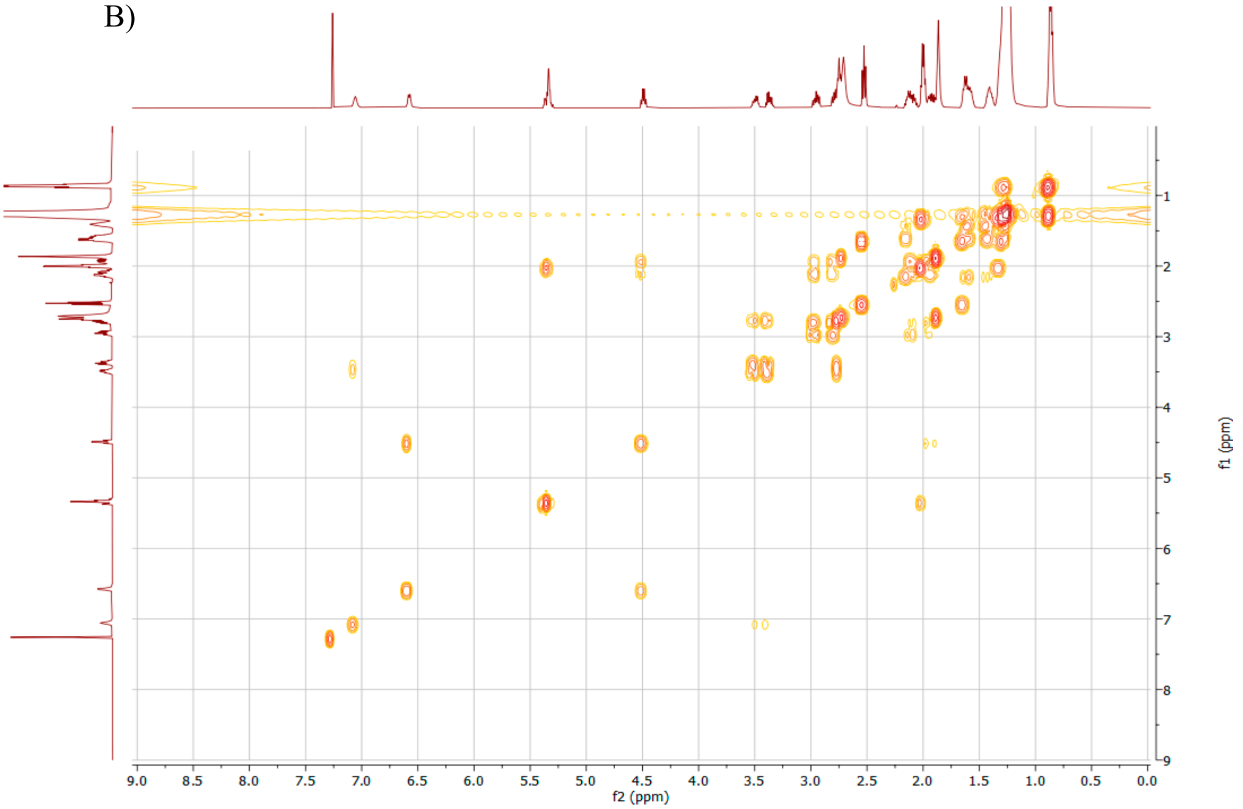

Figure S2. (A) ^{1}H-NMR spectra (CDCl3, 500 MHz) of ionizable lipid CP-LC-1539 with peak assignment. **(B)** 2D COSY spectrum of ionizable lipid CP-LC-1539.

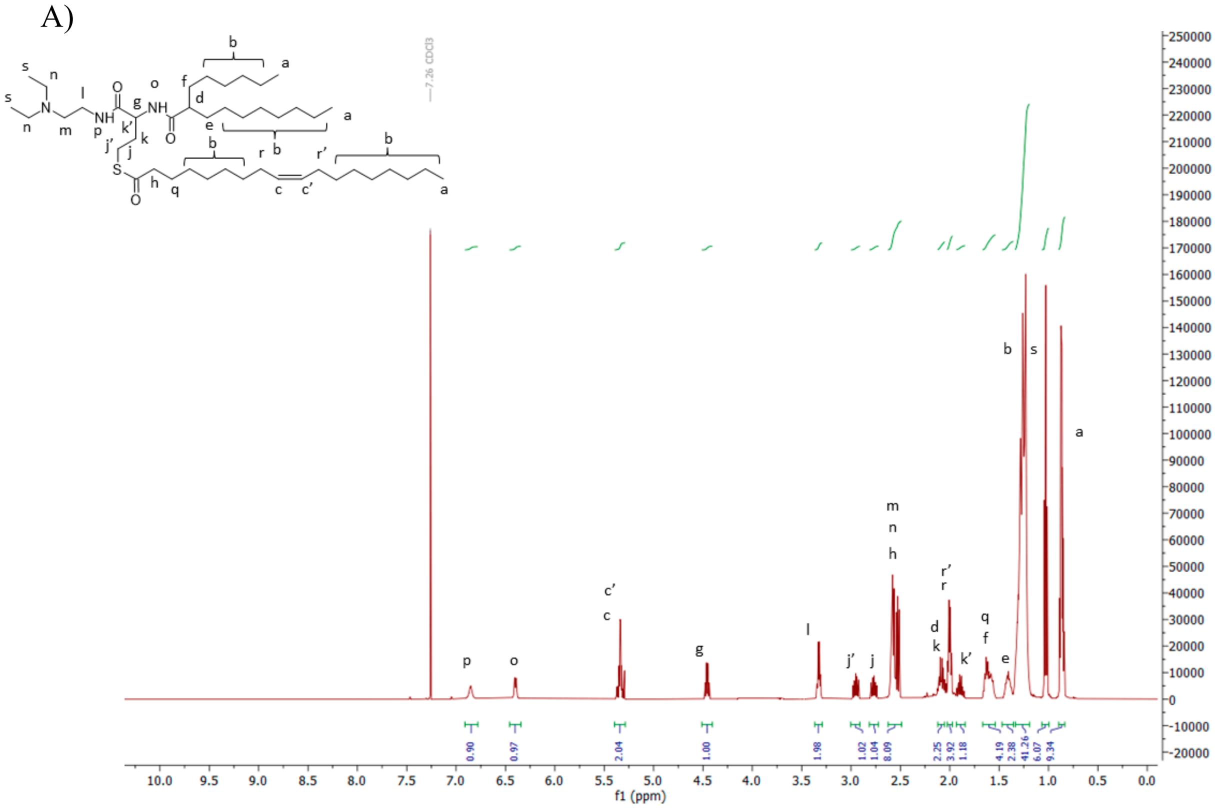

B)

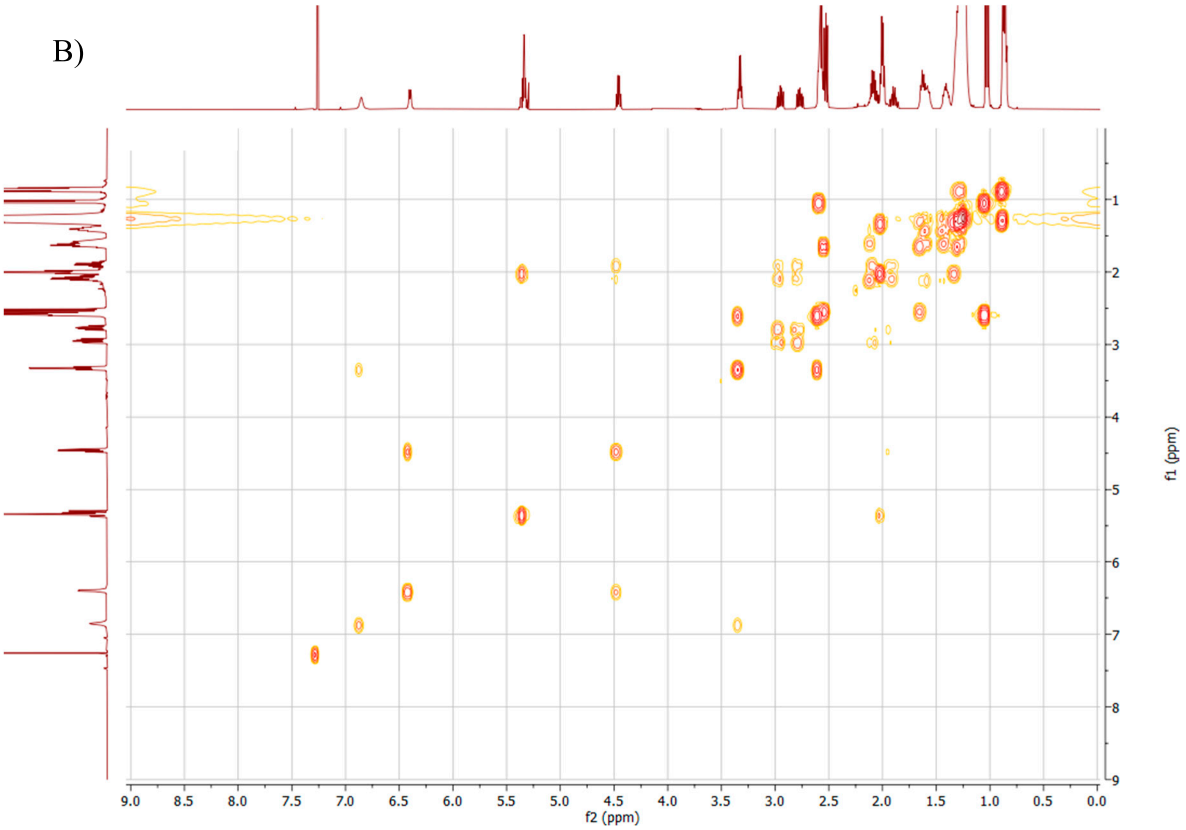

Figure S3. (A) ^{1}H-NMR spectra (CDCl3, 500 MHz) of ionizable lipid CP-LC-1545 with peak assignment. **(B)** 2D COSY spectrum of ionizable lipid CP-LC-1545.

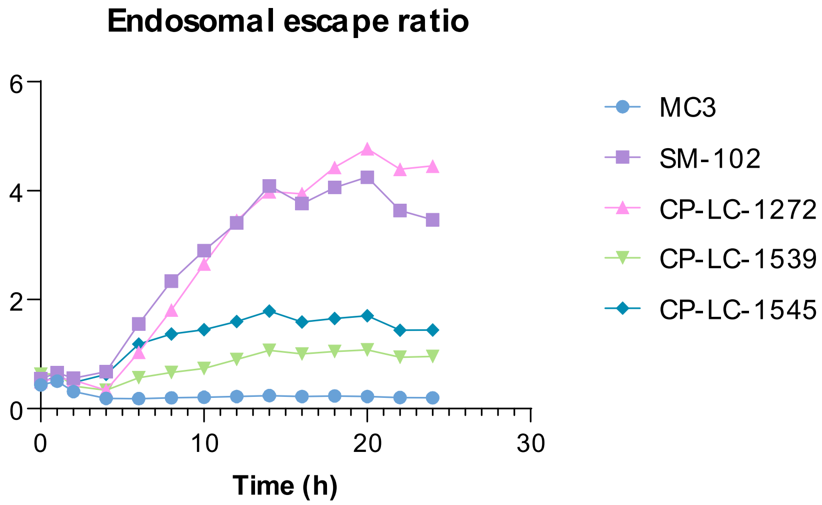

Figure S4. Endosomal escape efficiency is determined as the ratio of GFP expression to the fluorescence intensity of internalized LNPs.

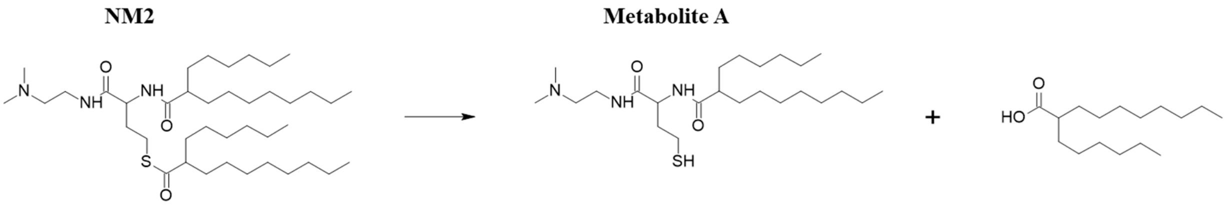

Figure S5. Metabolic products resulting from the thioester bond hydrolyzation of NM2 (CP-LC-1284).

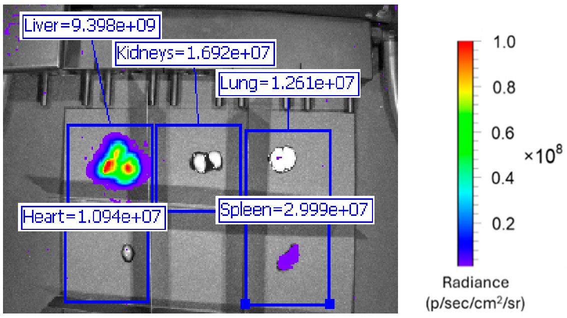

Figure S6. Representative *ex vivo* bioluminescence image of mouse organs employing CP-LC-1272 LNP encoding firefly luciferase mRNA.

Table S1. Characterization of formulated LNPs.

	Particle size (nm)	P.D.I.	Zeta potential (mV)	Apparent pKa	%EE
NS1	116.6	0.23	-3.5	7.35	97.34
NS2	123.2	0.21	-16.2	6.57	82.90
NM1 (CP-LC-1272)	119.60	0.17	3.7	7.01	97.23
NM2	93.74	0.17	-9.2	6.45	92.62
NL1	120.8	0.16	-9.4	6.92	91.23
NL2	105.6	0.19	-14.7	6.24	64.06
OS1	102.7	0.27	-3.5	7.11	99.64
OS2	98.89	0.16	-8.0	6.08	95.13
OM1	82.09	0.23	-10.5	6.17	92.23
OM2	86.13	0.18	-13.7	6.70	88.72
OL1	87.95	0.23	-20.1	6.10	86.90
OL2	83.35	0.17	-5.2	6.80	89.80
CP-LC-1539	125.4	0.37	9.1	6.70	90.41
CP-LC-1540	98.16	0.13	-1.8	6.41	91.56
CP-LC-1541	123.2	0.13	-5.1	6.47	84.88
CP-LC-1543	104.8	0.40	2.3	6.62	95.75
CP-LC-1545	123.1	0.065	1.5	6.60	93.12

LNPs were formulated by microfluidic mixing. The resulting lipid particles encapsulating Luc-mRNA were dialyzed overnight against a pH 8 Tris buffer solution containing 15% sucrose. Lipid particles were characterized by measuring their size distribution, polydispersity and Z-potential by dynamic light scattering (DLS). mRNA encapsulation was assessed by Quant-IT® Ribogreen following the manufacturer's instructions. Apparent pKa was calculated using a 6-(p-toluidinyl) naphthalene-2-sulfonic acid (TNS) assay.

Table S2. Components of LNPs in tRNA delivery assays.

	Components[a]	Molar ratio	N/P ratio (mol/mol)
MC3	MC3/DOPE/Chol/DMG-PEG/DiD'	50:10:38.5:1.5:0.5	6
SM-102	SM-102/DOPE/Chol/DMG-PEG/DiD'	50:10:38.5:1.5:0.5	6
CP-LC-1539	CP-LC-1539/DOPE/Chol/DMG-PEG/DiD'	50:10:38.5:1.5:0.5	6
CP-LC-1272	CP-LC-1272/DOPE/Chol/DMG-PEG/DiD'	50:10:38.5:1.5:0.5	6
CP-LC-1545	CP-LC-1545/DOPE/Chol/DMG-PEG/DiD'	50:10:38.5:1.5:0.5	6

[a)] DiD' (1,1'-dioctadecyl-3,3,3',3'-tetramethylindodicarbocyanine, 4-chlorobenzenesulfonate salt) was employed as a fluorescent label to assess cellular uptake

Table S3. Characterization of LNPs encapsulating GFP mRNA.

	Particle size (nm)	P.D.I.	Zeta potential (mV)	%EE
MC3	107.9	0.071	-13.5	86.80
SM-102	120.1	0.14	-8.7	94.36
CP-LC-1539	96.95	0.053	-5.0	98.09
CP-LC-1272	93.26	0.12	-10.1	95.22
CP-LC-1545	112.8	0.078	-9.3	95.31

LNPs were formulated by microfluidic mixing. The resulting lipid particles encapsulating GFP mRNA were dialyzed overnight against a pH 8 Tris buffer solution containing 15% sucrose. Lipid particles were characterized by measuring their size distribution, polydispersity and Z-potential by dynamic light scattering (DLS). mRNA encapsulation was assessed by Quant-IT® Ribogreen following the manufacturer's instructions.

Table S4. Endosomal escape ratio at 20 hours.

MC3	SM-102	CP-LC-1272	CP-LC-1539	CP-LC-1545
0.22	4.25	4.77	1.07	1.70

Table S5. Components of LNPs in tRNA delivery assays.

	RNA	Components	Molar Ratio	N/P ratio (w/w)
-tRNA LNP/PCT-mRNA LNP	mRNA	SM-102/DOPE/Chol/DMG-PEG	50:10:38.5:1.5	10
SM-102 tRNA LNP	tRNA	SM-102/DOPE/Chol/DMG-PEG	50:10:38.5:1.5	10
CP-LC-1272 tRNA LNP	tRNA	CP-LC-1272/DOPE/Chol/DMG-PEG	50:10:38.5:1.5	10
CP-LC-1545 LNP	tRNA	CP-LC-1545/DOPE/Chol/DMG-PEG	50:10:38.5:1.5	10

Table S6. Characterization of LNPs for tRNA delivery.

	Particle size (nm)	P.D.I.	Zeta potential (mV)	%EE
-tRNA LNP/PCT-mRNA LNP	102.7	0.11	-2.9	95.58
SM-102 tRNA LNP	116.3	0.24	-3.2	97.94
CP-LC-1272 tRNA LNP	104.1	0.078	-5.5	97.13
CP-LC-1545 LNP	138.3	0.23	-5.7	95.92

LNPs were formulated by microfluidic mixing. The resulting lipid particles encapsulating GFP mRNA were dialyzed overnight against a pH 8 Tris buffer solution containing 15% sucrose. Lipid particles were characterized by measuring their size distribution, polydispersity and Z-potential by dynamic light scattering (DLS). mRNA encapsulation was assessed by Quant-IT® Ribogreen following the manufacturer’s instructions.
